# Dopamine, Affordance and Active Inference

**DOI:** 10.1371/journal.pcbi.1002327

**Published:** 2012-01-05

**Authors:** Karl J. Friston, Tamara Shiner, Thomas FitzGerald, Joseph M. Galea, Rick Adams, Harriet Brown, Raymond J. Dolan, Rosalyn Moran, Klaas Enno Stephan, Sven Bestmann

**Affiliations:** 1 The Wellcome Trust Centre for Neuroimaging, University College London, Queen Square, London, United Kingdom; 2 Sobell Department of Motor Neuroscience and Movement Disorders, University College London Institute of Neurology, Queen Square, London, United Kingdom; Indiana University, United States of America

## Abstract

The role of dopamine in behaviour and decision-making is often cast in terms of reinforcement learning and optimal decision theory. Here, we present an alternative view that frames the physiology of dopamine in terms of Bayes-optimal behaviour. In this account, dopamine controls the precision or salience of (external or internal) cues that engender action. In other words, dopamine balances bottom-up sensory information and top-down prior beliefs when making hierarchical inferences (predictions) about cues that have affordance. In this paper, we focus on the consequences of changing tonic levels of dopamine firing using simulations of cued sequential movements. Crucially, the predictions driving movements are based upon a hierarchical generative model that infers the context in which movements are made. This means that we can confuse agents by changing the context (order) in which cues are presented. These simulations provide a (Bayes-optimal) model of contextual uncertainty and set switching that can be quantified in terms of behavioural and electrophysiological responses. Furthermore, one can simulate dopaminergic lesions (by changing the precision of prediction errors) to produce pathological behaviours that are reminiscent of those seen in neurological disorders such as Parkinson's disease. We use these simulations to demonstrate how a single functional role for dopamine at the synaptic level can manifest in different ways at the behavioural level.

## Author Summary

Dopamine is a neurotransmitter that has been implicated in a wide variety of cognitive and motor functions; it is depleted in Parkinson's disease, disrupted in schizophrenia and plays a central role in working memory, reinforcement learning and other cognitive functions. In this paper, we present a straightforward and neurophysiologically grounded explanation for the diversity of functions and pathologies that implicate dopamine. This explanation rests on a principled approach to the nature of action and perception called active inference. This approach suggests that (Bayes) optimal perception and consequent behaviour depends on representing uncertainty about states of the world in terms of the precision (inverse amplitude) of their random fluctuations. Crucially, this uncertainly can be encoded by the same postsynaptic gain of neurons that is modulated by dopamine. This means that changing the levels of dopamine changes the level of uncertainty about different representations. To substantiate this idea, we simulate dopamine depletion in a hierarchical sensorimotor network to show that a single function of dopamine (encoding precision in terms of postsynaptic gain) is not only sufficient to account for commonly observed behaviours following dopamine depletion but also provides a unifying perspective on many existing theories about dopamine.

## Introduction

This article is about set switching and action selection during the execution of cued responses. It offers a straightforward account of dopamine in optimising behaviour in the context of (Bayes-optimal) predictive coding. Our focus is on the consequences of depleting dopamine and simulating perseveration that is characteristic of Parkinson's disease, using synthetic neuronal models based upon *active inference* [1]. In brief, the emergent role of dopamine is to report the precision or salience of perceptual cues that portend a predictable sequence of sensorimotor events. In this sense, it mediates the affordance of cues that elicit motor behaviour [2]; in much the same way that attention mediates the salience of cues in the perceptual domain. Gibson defined affordances as *action possibilities* latent in the environment [3], objectively measurable and independent of the ability to recognize them but always in relation to the actor [4]. Affordance is therefore an attribute of a cue and has to be inferred. Crucially, in this paper, inferring that an object has affordance necessarily entails an action. We hope to establish a central role for dopamine in this inference and implicit action selection.

Dopamine has been implicated in a bewildering variety of processes and pathologies in the human brain; ranging from cortical excitability to attentional deficits [5], [6]; from motor control to akinesia and set switching deficits in Parkinson's disease [7], [8], [9]; from working memory to schizophrenia [10], [11]; from reinforcement learning to addiction [12], [13]; from executive function to age-related cognitive decline [14]; from reward prediction to failures of incentive salience [15], [16]; from exploration to psychomotor poverty [17]. In fact, it is difficult to find an area of neuroscience that does not implicate dopamine; for example, it has key roles in mood, sleep, nociception, and prolactin production. In terms of its functional or computational roles, it has been suggested that dopamine reports reward prediction errors, hedonic value, incentive salience, novelty, and so on. Many accounts appeal to optimal decision theory and reinforcement learning to understand the putative role of dopamine in formal terms [18], [19], [20], [21]. These treatments rest on the assumption that behaviour is optimal in relation to some reward or cost function and invoke various heuristics from control theory (e.g., dynamic programming) to explain the computational role of dopamine.

This article takes a different view and considers that behaviour is Bayes-optimal in the sense that it maximises the Bayesian evidence for an actor's model of the world or, equivalently, minimises surprise. We have formulated this as *active inference* in a series of previous papers [1], [22], [23]. Active inference can be seen as an embodied (enactivist) form of predictive coding, in which perception minimises exteroceptive prediction errors and action minimises proprioceptive prediction errors. Put simply, active inference is predictive coding with classical motor reflexes. In this setting, cost functions are replaced by surprise or prediction error, in the sense that the only optimal behaviour is a behaviour that brings about expected outcomes (i.e., minimises surprise as opposed to cost). This ensures that agents avoid potentially harmful or surprising exchanges with the environment and equips them with a physiological and ethological homoeostasis. Note that this does impose constraints on behaviour, since appropriate priors can replicate the effect of any cost function [1]. In short, rewards are just familiar sensory states. This perspective has three advantages over reward-based accounts: first, it resolves the tautology inherent in the notion of reward. This tautology follows from the fact that reward is used to explain reward seeking behaviour [24], while at the same time being defined in terms of its ability to elicit reward seeking behaviour. Second, it dispenses with the intractable solutions of control theory problems (e.g., Bellman optimality equations) that are necessary to optimise reward or cost functions [25], [26], [27]. Finally, and central to this paper, it provides a novel perspective on the role of dopamine that accounts for its apparently diverse roles in terms of a single mechanism, operating at different levels of the sensorimotor hierarchy.

The Bayesian perspective suggests that there are only two sorts of things that need to be inferred about the world; namely, the state of the world and uncertainty about that state. We have suggested that predicted states of the world are encoded in terms of synaptic activity, while uncertainty is encoded by synaptic gain that encodes the precision (inverse amplitude or variance) of random fluctuations about predicted states [28]. If true, this means that modulators of synaptic gain (like dopamine) do not report perceptual content but the context in which percepts are formed. In other words, dopamine reports the precision or salience of sensorimotor constructs (representations) encoded by the activity of the synapses they modulate. This leads to a view of dopaminergic projections that select salient processing channels and associated actions. Physiologically, this is compatible with short latency dopamine bursts in the basal ganglia that occur after any salient event, whether rewarding or not [29]. In this view, dopaminergic discharges do not signify reward prediction errors but are an integral part of Bayes-optimal perception and sensorimotor integration: they respond to salient or precise cues that portend a predictable sequence of sensorimotor events that will be registered by specific proprioceptive and exteroceptive processing channels. Crucially, these responses will appear to be reward-related, because they precede sensorimotor sequences that lead to a rewarding (familiar) state. In other words, if a sequence of choices is predictable they lead to unsurprising outcomes, which are, by definition, rewarding. However, the agent may have no concept or representation of reward; it is just doing what it expects to do in the context established by the pattern of dopamine firing.

The motivation and mechanisms behind the role of dopamine considered in this paper are exactly the same as we have proposed for the attentional modulation of postsynaptic gain in sensory processing [30]. Both attentional modulation and dopaminergic gating may represent a Bayes-optimal encoding of precision that enhances the processing of particular sensory representations by selectively biasing bottom-up sensory information (prediction errors). In other words, it confers salience on attended representations [31], [32], [33], [34], [35], [36], [37]. The specific role of dopaminergic neurotransmission in behaviour (as opposed to perception) may be explained by the regional specificity of its projection fields (and postsynaptic receptor subtypes) that are mainly confined to cortical and subcortical structures concerned with predicting choices and motor responses [38]. This is important because it means that dopamine may be exclusively concerned with salient representations that have affordance; in other words, sensorimotor representations that predict both perceptual and behavioural consequences. If true, this means dopamine has a crucial role in biasing sensorimotor integration and action selection. More formally, dopamine is in a position to select the proprioceptive and exteroceptive signals (prediction errors) that compete for higher level explanation by controlling their precision. This formulation sits comfortably with the affordance competition hypothesis [2], [39] and other theoretical accounts: for example, the uncertainty processing theory of motivation [40], neurobiological accounts of decision-making [41], [42] and the plurality of roles suggested by the physiology of dopamine [43]. In particular, it draws on the same notions that link dopamine to the encoding of uncertainty [44] and adaptive responses to changes in neuronal signal to noise levels [45], [46]. This is because precision represents uncertainty due to random fluctuations or noise. By associating salience with precision we can also connect to constructs like *incentive salience* in psychology [47] and *aberrant salience* in psychopathology [48]. Indeed, it has been shown that action selection can be cast as signal selection using salience to report the “propensity for selecting a given action” [49]. Note that precision or salience is an attribute of a (probabilistic) representation that determines the confidence or certainty about what is represented; where the salience of sensory representations can be manipulated experimentally, by changing signal to noise levels or contrast. We will see an example of this later. Finally, the notion that dopamine modulates synaptic gain plays a key role in several proposals. It has been argued for in [50] and used in several Parkinson's disease modelling papers [51], [52].

In this paper, the hypothesis that dopamine release reports precision or uncertainty is based purely on its synaptic physiology. However, there is definitive neurophysiological evidence for this role of dopamine [44], where, for example, dopaminergic discharges covary with the variance or precision of juice rewards [53]. More generally, nearly every experimental manipulation evoking dopaminergic responses (novelty, unexpected rewards, etc) speaks to a change in the level of precision or confidence about subsequent contingencies. In what follows, we try to substantiate the above ideas using theoretical arguments based upon active inference and then illustrate their plausibility using simulations of cued responses. These simulations are concerned with the consequences of depleting dopaminergic neurotransmission to illustrate its central role in action selection and set switching. They can therefore be regarded as a very simple model of Parkinson's disease. This means we will not address changes in precision but assume that the tonic activity of dopaminergic neurons encodes a fixed level of precision or uncertainty [44], [43], [45]. Subsequent papers will focus on the control of (phasic and tonic) dopaminergic responses *per se* and will try to reproduce the empirical findings of behavioural reinforcement paradigms, using phasic dopamine discharges that shift striatial neurons into an *up state* to increase their gain or precision [54]. Furthermore, we will restrict our discussion to the generic effect of dopamine on postsynaptic D1 receptors located on principal cells throughout the brain [55]. This necessarily precludes a proper consideration of the balance between D1 and D2 receptor function and its relationship to the functional anatomy of the basal ganglia in Parkinson's disease [56].

The models and methods section reviews the theory on which subsequent simulations are based. This section presents a brief review of the free energy principle, with a special focus on active inference and the role of synaptic gain in encoding precision. The basic theory and ensuing differential equations used to simulate neuronal responses are exactly the same as those used to illustrate perceptual inference, learning, attention and action in a series of previous papers (Table 1). This formalism is then used to model sequential cued movements, under normal and, in the results section, depleted levels of dopamine. The particular simulations used in this paper rest on prior beliefs about sensorimotor trajectories, encoded by itinerant (wandering) dynamics in premotor cortex. These dynamics are entrained by prediction errors from the superior colliculus, the parietal cortex and motor cortex, whose precision is, we assume, controlled by dopamine. Using this architecture, we can simulate visually cued sequences of movements and, crucially, responses to sequence violations. By adding a further (prefrontal) level to the model, we examine how the ability to switch from one sequence to another is compromised when sensorimotor cues lose precision. The resulting impact on set switching is characterised in terms of (synthetic) neuronal responses and behavioural (reaction time and accuracy) measures. We conclude with a discussion of the implications for dopamine in motor control and set switching generally, and for Parkinson's disease specifically.

**Table 1:** Processes and paradigms that have been modeled using the scheme in this paper.

Domain	Process or paradigm
Perception	*Perceptual categorization (bird songs)* [70]
	*Novelty and omission-related responses* [70]
Sensory learning	*Perceptual learning (mismatch negativity)* [139]
Attention	*Attention and the Posner paradigm* [30]
	*Attention and biased competition* [30]
Motor control	*Retinal stabilization and oculomotor reflexes* [22]
	*Saccadic eye movements and cued reaching* [22]
	*Motor trajectories and place cells* [23]
Sensorimotor integration	*Bayes-optimal sensorimotor integration* [22]
Behavior	*Heuristics and dynamical systems theory* [140]
	*Goal-directed behavior* [1]
Action observation	*Action observation and mirror neurons* [23]

## Methods

### Active inference, affordance and free energy

In this section, we briefly overview the free energy principle and active inference to frame the role of dopamine examined later. The free energy principle proposes that the states and infrastructure of a self organising system, such as the brain, should minimise the free energy of the sensory states it samples [57]. Free energy is an upper bound on the surprise associated with sensory signals, where surprise is mathematically the same as the (negative log) Bayesian evidence for the system's model of its world. Evidence is just the probability of getting some data under the model of those data. This means that minimising free energy reduces surprising exchanges with the environment or, equivalently, maximises the evidence for an agent's internal model of its sensorium. This principle entails two corollaries; the Bayesian brain hypothesis [58], [59]; [60], [61] and active inference [1], [22], [23]. The Bayesian brain hypothesis means that the brain will try to predict its sensory inputs in a Bayes-optimal fashion by representing their causes in terms of hidden states of the world. Active inference equips the Bayesian brain with motor reflex arcs that ensure its predictions are fulfilled (by suppressing proprioceptive prediction errors). In active inference, behaviour emerges as natural consequence of high-level representations (sensorimotor constructs) that have both sensory (exteroceptive) and motor (proprioceptive) consequences. These constructs or representations are maintained by bottom-up prediction errors in both modalities and reciprocate top-down (proprioceptive) predictions to the peripheral motor system that drive classical motor reflexes; while top-down predictions to sensory systems play the role of corollary discharge and suppress (exteroceptive) prediction errors.

Crucially, high-level sensorimotor representations can be dynamic in nature, with itinerant dynamics (on attractor manifolds) that embody prior beliefs about the sequence of sensorimotor events or trajectories that will unfold in the near future. These can be regarded as central pattern generators or attractors that provide proprioceptive and sensory predictions for sensorimotor integration; in other words, representations of affordance [23]. The particular sequence currently active depends upon which attractor has been selected. This selection rests upon precise bottom-up prediction errors conveying salient sensory information that has yet to be explained. In this scheme, prediction errors can induce or destroy metastable attractors at higher levels to select the trajectory that best explains sensory input. This can be regarded as selecting an attractor with an affordance that best explains sensory input; cf., “affordance competition” in [2]. The potency with which ascending prediction errors can select the appropriate attractor depends upon their postsynaptic gain. This gain encodes the precision (inverse variance) of random fluctuations about predictions. In other words, the ability of bottom-up prediction errors to bias competition among high level sensorimotor representations (attractors) depends upon their precision that we presume, in this paper, is modulated by dopamine. In the results section, we will see an example of these dynamics and what happens when the precision of prediction errors is reduced by (simulating) a reduction in dopaminergic neurotransmission. We hypothesised that this would result in a failure of set switching and the perseveration of sensorimotor dynamics of the sort seen in Parkinson's disease [7], [62], [63]. In what follows, we will unpack the above summary in slightly more formal terms:

### Generative models and the Bayesian brain

The equations and simulations used in this paper may appear a bit complicated and *ad hoc*; however, they are based on just three assumptions:

- The brain minimises the free energy of sensory inputs defined by a generative model.
- The generative model used by the brain is hierarchical, nonlinear and dynamic.
- Neuronal firing rates encode the most likely state of the world, under this model.

The first assumption is the free energy principle, which leads to active inference when considering both representations and action. This principle has to be true, in the sense that a failure to minimise free energy means that the brain will entertain increasingly surprising sensations and, at some point, will cease to exist in an ergodic sense. The second assumption is motivated easily by noting that the world is both dynamic and nonlinear. Hierarchical causal structure emerges inevitably from a separation of temporal scales. This can be seen most clearly in the *slaving principle* from statistical physics [64], [65], where slow ordered dynamics emerge at a macroscopic scale and enslave fast fluctuations at a microscopic scale. Finally, the third assumption follows from the first, under the constraint that probabilistic representations are encoded by a minimum number of biophysical variables. This leads to something called the Laplace assumption, in which the probability density function over hidden states is Gaussian and can be encoded by its mean or expectation. In terms of neural codes, this is referred to this as the *Laplace code* and is arguably the simplest and most flexible of all neural codes [66].

Given these three assumptions, one can simulate a whole variety of situations and processes by simply specifying the particular equations that constitute the generative model. The resulting perception and action is specified completely by the above assumptions and can be implemented in a biologically plausible way as described in previous applications listed in Table 1. In brief, these simulations use differential equations that minimise the free energy of sensory input using a generalised gradient descent [67].
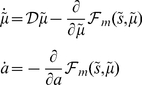
(1)These coupled differential equations describe perception and action respectively and just say that internal brain states and action change in the direction that reduces free energy. The first is known as (generalised) predictive coding and has the same form as Bayesian (e.g., Kalman-Bucy) filters used in time series analysis; see also [68]. The free energy 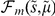 depends on three things; sensory signals 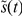, conditional expectations or representations 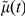 and a model 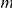. The model defines how expectations about states of the world conspire to produce sensory input. In neurobiological formulations, these expectations or predictions are associated with neuronal activity and the model comprises a connectivity or network architecture. The∼notation denotes variables in generalised coordinates of motion that include velocity, acceleration, jerk and so on; 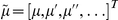. The first term in Equation 1 is a prediction based upon a matrix differential operator 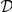 that returns the generalised motion of the expectation, such that 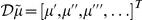. The second term is usually expressed as a mixture of prediction errors that ensures the changes in conditional expectations are Bayes-optimal predictions about hidden states of the world. The second differential equation says that action also minimises free energy. The differential equations are coupled because sensory input depends upon action, which depends upon perception through the conditional expectations. This circular dependency leads to a sampling of sensory input that is both predicted and predictable, thereby minimising free energy and surprise.

To perform neuronal simulations under this framework it is only necessary to integrate or solve Equation 1 to simulate neuronal dynamics that encode the conditional predictions and ensuing action. Conditional predictions depend upon the brain's generative model of the world, which we assume has the following (hierarchical) form
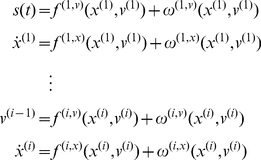
(2)This equation is just a way of writing down a model that relates various quantities in the world probabilistically in terms of their generalised motion. Here, 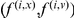 are nonlinear functions of hidden states and causes 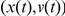 that generate sensory inputs 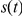 at the first (lowest) level. Random fluctuations 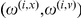 on the motion of hidden states and causes are conditionally independent and enter each level of the hierarchy. It is these that make the model probabilistic. They play the role of sensory noise at the first level and induce uncertainty about states at higher levels. The (inverse) amplitudes of these random fluctuations are quantified by their precisions; 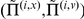, which we assume to be fixed in this paper and encoded by dopamine. Hidden causes 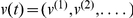 link hierarchical levels, whereas hidden states 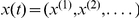 link dynamics over time. Hidden states and causes are abstract quantities (like the motion of an object in the field of view) that the brain uses to explain or predict sensations. In this hierarchical model, the output of one level acts as an input to the next. This input can produce complicated (generalised) convolutions with deep (hierarchical) structure. We will see an example of this later.

### Perception and action under predictive coding

Given the form of the generative model (Equation 2) we can now write down the differential equations (Equation 1) describing neuronal dynamics in terms of (precision-weighted) prediction errors on the hidden causes and states 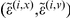. These errors represent the difference between conditional expectations 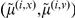 and predicted values, under the generative model:
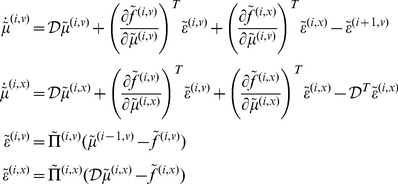
(3)At the lowest level of the hierarchy, conditional expectations are replaced by sensory input. This equation can be derived fairly easily by computing the free energy for the hierarchical model in Equation 2 and inserting its gradients into Equation 1. What we end up with is a relatively simple update scheme, in which conditional expectations are driven by a mixture of prediction errors, where prediction errors are defined by the equations of the generative model.

It is difficult to overstate the generality and importance of Equation 3: its solutions grandfather nearly every known statistical estimation scheme, under parametric assumptions about additive or multiplicative noise [69]. These range from ordinary least squares to advanced variational deconvolution schemes. The resulting scheme is called *generalised filtering* or generalised predictive coding [67]. In neural network terms, Equation 3 says that error-units receive predictions from the same level and the level above. Conversely, prediction-units are driven by prediction errors from the same level and the level below. These constitute bottom-up and lateral messages that drive conditional expectations towards a better prediction to reduce the prediction error in the level below. This is the essence of recurrent message passing between hierarchical levels to optimise free energy or suppress prediction error: see [70], for a more detailed discussion. In neurobiological implementations of this scheme, the sources of bottom-up prediction errors, in the cortex, are thought to be superficial pyramidal cells that send forward connections to higher cortical areas. Conversely, predictions are conveyed from deep pyramidal cells, by backward connections, to target (polysynaptically) the superficial pyramidal cells encoding prediction error [71], [70]. The laminar specificity of the cells of origin of predictions and prediction errors becomes relevant when examining the putative role of dopamine in the encoding of precision.

### The neurobiology of precision

Equation 3 shows that precision modulates the responses of prediction error units to their presynaptic inputs. Here, we associate precision with dopaminergic neuromodulation of these responses. The action of dopamine is mediated by a family of transmembrane G protein-coupled receptors [72] encoded by at least five dopamine receptor genes [73]. Dopamine receptors are found throughout the soma and dendrites of neurons but ultra-structural and biochemical evidence suggests that they are concentrated in dendritic spines that express glutamatergic synapses [74], [75]. Postsynaptic D1 and D2 receptors are therefore strategically positioned to control the excitability and synaptic properties of spines “with remarkable precision and versatility” [76].

Although dopamine appears to be a natural candidate to modulate principal cells reporting prediction error, Equation 3 does not tell us whether prediction errors are modulated before or after they are computed. Both mechanisms are biologically plausible: for example, superficial pyramidal cells encoding prediction error could be modulated by D1 receptors on the soma or initial segment, after the integration of signals subtending prediction errors in the dendritic tree. Conversely, precision dependent modulation could be applied at a synaptic level to all presynaptic inputs. For dopamine, the balance of evidence points to the latter mechanism [77]:

Dopamine innervation in the human prefrontal cortex exhibits a distinct bilaminar distribution with dense bands of fibres in the superficial and deep layers [78], [74]. Although some evidence suggests that dopaminergic markers are more concentrated in deep layers [79], [80], other studies report a higher concentration in supragranular layers [81]. These differences may be due to regional and species differences [82]. Here, we will focus on dopaminergic modulation of cells in the supragranular layers, because superficial pyramidal cells are thought to report prediction error [71]. Dopamine axons form symmetric synapses, predominantly on the spines of pyramidal cells. In many cases, the same spine expresses an asymmetric (excitatory) synapse. In human and monkey prefrontal cortex, the dopamine D1-specific ligand, 3H-SCH23390, and the D2-specific ligand, H3-raclopride, label binding sites that mirror the densest dopamine innervation [78], [83]. This suggests that the primary role of D1 receptors is to modulate presynaptic input to pyramidal cells at the dendritic level [77], [74]. In terms of the implicit computational architecture, we can therefore assume that dopamine gates or modulates the dendritic responses of superficial pyramidal cells, such that dopamine selects afferents encoding sensory information (prediction error) in proportion to its precision.

**Figure 1:**
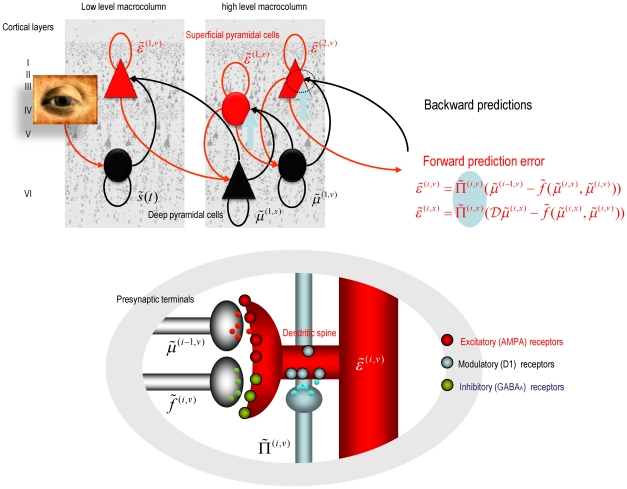
This figure provides a schematic overview of the message passing scheme implied by **Equation 3**. In this scheme, neurons are divided into prediction (black) and prediction error (red) units that pass messages to each other, within and between hierarchical levels. Superficial pyramidal cells (red) send forward prediction errors to deep pyramidal cells (black), which reciprocate with predictions that are conveyed by (polysynaptic) backward extrinsic connections. This process continues until the amplitude of prediction error has been minimized and the predictions are optimized in a Bayesian sense. The prediction errors are the (precision weighted) difference between conditional expectations encoded at any level and top down or lateral predictions. Note that there are prediction errors at every level of the hierarchy, for both hidden states and hidden causes (and sensory states and the lowest level). The synaptic infrastructure proposed to mediate this comparison and subsequent modulation is shown in the lower panel, in terms of a doubly-innervated synapse [84] that is gated by dopamine (cyan). Here, dopamine is delivered by *en passant* synaptic boutons and postsynaptic D1 receptors have been located on a dendritic spine expressing asymmetric (excitatory) and symmetric (inhibitory) synaptic connections. This represents the synaptic arrangements indicated by the cyan arrows in the upper panel.

Figure 1 provides a schematic of the neuronal circuitry implied by this assumption, in which dopamine modulates doubly-innervated spines of superficial pyramidal cells receiving excitatory and inhibitory presynaptic inputs [84]; corresponding to conditional expectations and their predictions respectively. The opposing effects of these presynaptic inputs on postsynaptic depolarisation form a prediction error signal that is modulated at the level of the dendritic spine by dopamine. In this scheme, the tonic firing of a particular dopaminergic cell or population encodes the precision or salience of the information (prediction error) conveyed by the cells that it targets. One might imagine that phasic discharges report changes in the current context and signal a change in the relative precision (uncertainty) over different sensory channels and conditional predictions. In this paper, we will assume that the precision at each hierarchical level is constant and address the (phasic) control of dopaminergic activity in terms of optimisation of (state-dependent) precision elsewhere.

### Action and affordance

Clearly, in sensorimotor hierarchies [85], the relative levels of neuromodulatory gating (precision) at different levels can have a profound effect on perception and behaviour, because it will select particular processing channels and change the balance between bottom-up sensory information and top-down prior expectations. In hierarchical models, these prior expectations are called *empirical* priors, because they are optimised in relation to sensory data. Furthermore, precision or neuromodulation of synaptic gain will affect action and motor control, because action is driven by (proprioceptive) prediction errors at the sensory level that have their own gain or precision:
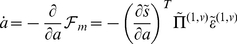
(4)This follows because the only way that action can minimise free energy is to change sensory prediction errors by selecting which sensory signals are sampled. As noted above, the ensuing suppression of proprioceptive prediction errors can be thought of in terms of classical motor reflex arcs: see [22] for details.

Affordance is generally conceived of as the opportunities for action offered by the environment to an agent [4]. This depends on both the environment and the nature of the actor. For example, an axe only affords the possibility of use when it can be wielded. In this paper, affordance is an attribute of amodal representations at higher hierarchical levels that make both sensory and motor predictions (an ‘axe’ entails predictions not only about how it looks or feels, but also the kinaesthetic consequences of wielding it). Conditional expectations or representations with affordance elicit behaviour by sending top-down predictions down the hierarchy that are unpacked into proprioceptive predictions at the level of the cranial nerve nuclei and spinal-cord. These engage classical reflex arcs to produce the predicted motor trajectory. The action of dopamine in this context is to modulate or enable the salience of representations that have affordance, and hence the probability they will be enacted.

### Summary

In summary, we have derived equations for the dynamics of perception and action using a free energy formulation of adaptive (Bayes-optimal) exchanges with the world and a generative model that is both generic and biologically plausible. In what follows, we use Equations 3 and 4 to simulate neuronal and behavioural responses. A technical treatment of the material above will be found in [67], which provides the details of the scheme used to integrate (solve) Equation 1 to produce the simulations considered next.

### A generative model of cued responses

The preceding scheme allows one to simulate (Bayes-optimal) responses in terms of neuronal activity and motor behaviour, under any plausible generative model. Here, we consider a particular model, described in terms of the functions in Equation 2 that leads to a sequence of pointing movements, elicited by a sequence of visual cues. This model of sensorimotor integration provides the basis for simple simulated lesion experiments, in which we can deplete levels of simulated dopamine (precision) in different parts of the brain, and examine the consequences.

Because the differential equations governing perception and action are coupled (Equation 1), we need to specify two mappings: the generative model used by the brain, whose inversion maps from sensations to action, and the process by which action produces sensations. To distinguish between real states generating sensory information and the hidden states assumed by the generative model, we will use bold and italic variables respectively. Sensory input was generated using the following equations, which constitute real world dynamics that are hidden from the agent:
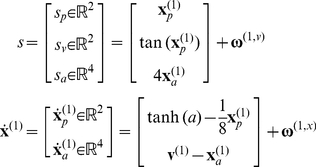
(5)This equation specifies how hidden states in the world produce sensory inputs and how those states change in response to action and hidden causes. Although it may look complicated, the implicit dynamics are very simple: The first equation expresses sensory input as a nonlinear function of true states in the world that comprise the position of a single jointed arm 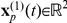 in terms of vertical and horizontal angles of displacement from a resting position 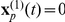; and the salience 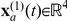 of four locations in extrinsic (visual) coordinates. Salience corresponds to appearance of visual cues at the four locations that affords the possibility of reaching towards them. These hidden states produce sensory signals in a proprioceptive modality, 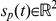; the location of the arm in visual coordinates, 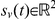 and the salience (e.g., illumination) of the four target locations 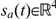. Here, we have used a simple tangent function to model the nonlinear transformation from intrinsic (proprioceptive) to extrinsic (visual) coordinates that is inherent in real motor control [86].

The second differential equation describes how changes in arm position and target salience depend upon action and exogenous causes respectively: the direction of the arm changes as a sigmoid (hyperbolic tangent) function of action and decays back to its resting position in the absence of action. The salience of each location is increased by exogenous causes 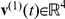 that we can use to specify the duration and sequence of (visual) cues. The motion of the states and sensory input were subject to low levels of noise in the simulations (with a log precision of 16).

Clearly, to integrate Equation 5 we need not only the exogenous causes (that specify sequence of visual cues) but also action that depends upon the perceptual inversion of a generative model. The generative model here was chosen to include several features of sensorimotor integration and hierarchical dynamics in the simplest way possible. It considers the brain to model the sensory world as a succession of unstable fixed points in some abstract state space. In other words, the agent expects the world to change continuously, with an itinerant (wandering) trajectory, visiting different states in succession. These reflect prior beliefs about forthcoming sensory events, and are encoded by differential equations that embody metastable dynamics; e.g., winnerless competition; [87], [88]. The resulting attractors can be thought of as central pattern generators that can be nested hierarchically at different time scales [89] to produce exteroceptive and proprioceptive (i.e., sensorimotor) predictions. These predictions are entrained by perception and prescribe motor responses through active inference. An important aspect of these generative models is that high level dynamics determine the context or set that engages lower-level sensorimotor sequences. In what follows, we will exploit this hierarchical aspect to illustrate some generic features of set switching and action selection and how they depend on the delicate balance of precision over different hierarchical levels.

The generative model used by our simulated agent had two levels and the following form (a particular case of Equation 2):
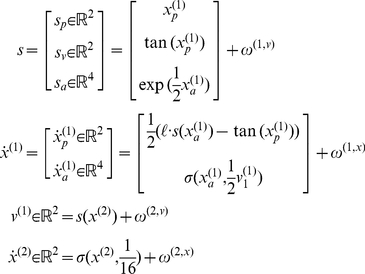
(6)At the sensory level, this model has the same form as the actual process generating sensory information (in Equation 5), with the exception that salience is log transformed so that it can be positive or negative (hence the exponential function in the first equality). However, above the sensory mapping (the first equation) the form of a model is very different from the process generating data (Equation 5) and embodies (formal) priors that induce a dynamic interplay between action and perception. It is this interplay that underlies the agent's behaviour. Crucially, the hidden states 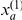 not only predict visual salience but also predict changes in proprioception. In this sense, they become affordance states. More specifically, the second differential equation means that the agent expects its arm to be drawn to the location specified by a function 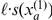 of hidden affordance states, where 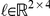 encodes the location of the four targets or cues. A softmax function of hidden affordance states 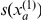 just ensures that the hidden state with the largest affordance predominates over the others. It is important to note that if we did not add anything else to this generative model, the agent would simply point to each target when it appeared; cf., [90]. This is because perception would infer that the affordance of a particular target was high. The associated conditional expectations would then induce proprioceptive predictions that would be fulfilled by action; such that the agent would point towards the target. In an experimental setting, these prior beliefs may be instantiated through task instructions.

**Figure 2:**
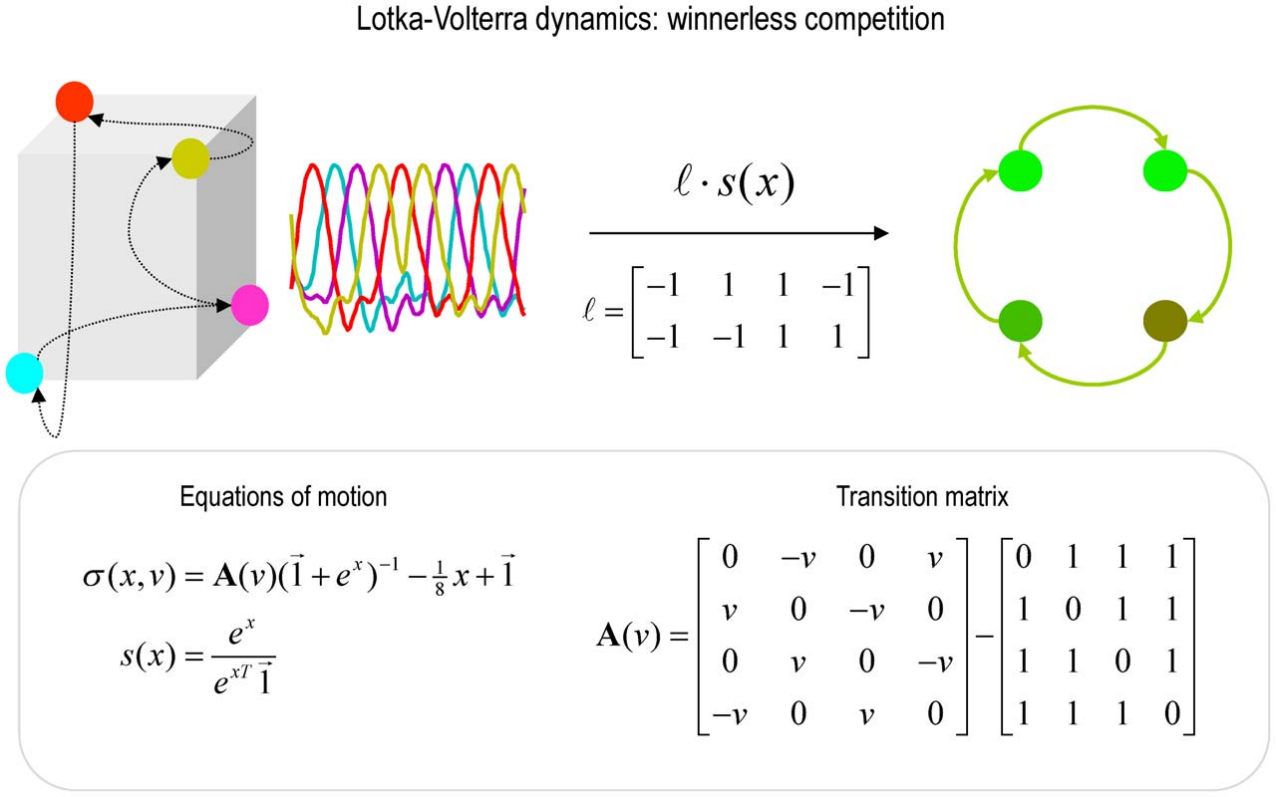
This figure provides a schematic overview of winnerless competition. These itinerant (wandering) dynamics are used to model sequential neuronal dynamics that, in this paper, encode prior beliefs about sequential changes in hidden states (e.g., affordance). Technically, these dynamics comprise stable heteroclinic channels or cycles that connect unstable fixed points. The fixed points are the colored dots in the upper left diagram. Each unstable fixed point is attractive in one dimension and repelling in another, expelling the state 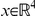 so that it is captured by the next unstable fixed point and so. A common example of these dynamics is provided by predator-prey relationships modeled with Lotka-Volterra *equations of motion*, denoted by 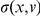 in the lower panel. The speed with which the fixed points are visited is controlled by a variable 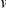 that scales the elements in a *transition matrix* 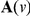, which couples the attractor states. In this paper, the attractor states are mapped to fixed locations in an extrinsic (physical) frame of reference to encode their affordance, using a softmax function of the attractor states 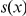 and a matrix 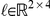, encoding their locations. This means that the orbit or trajectory in the four dimensional attractor space maps to a two-dimensional trajectory, which cycles through the four locations in a fixed order. We use this trajectory to generate forces that elicit pointing movements: See [87] and [23] for details.

However, we will consider a more sophisticated and realistic model, in which the agent has prior beliefs about the sequence in which targets will appear. This sort of prior belief could be instantiated by repeated exposure to the same sequence. These prior beliefs are encoded by the function 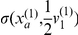 that prescribes a stable heteroclinic channel or winnerless competition among hidden affordance states. In other words, the agent believes that the affordance of the four target locations will change continuously, where each of the four states rises in turn, exciting the next state and suppressing itself (see Figure 2). The speed of these sequential dynamics is governed by the hidden cause 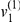. If we stopped here, we would have a simple agent with a limited repertoire of expectations that comprised a fixed sequence of sensorimotor events. These expectations may be consistent with the actual order of cues encountered or they may not be.

The final part of the model endows the agent with the concept that cues may or may not be ordered. We model this in terms of the hidden cause that controls the speed of the sequence. Crucially, this hidden cause is itself a softmax function of a hidden state that is part of a slower itinerant cycle (by factor of eight), governed by the same winnerless competition among the hidden states; 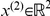. This means that, depending upon the second level hidden states; the sequential dynamics of the hidden affordances at the first level may or may not be engaged. The resulting model may sound complicated; however, its complexity lies in labelling various states of the model. The actual form of the model is both mathematically quite simple and biologically plausible: we have just placed a slow pattern generator on top of a fast pattern generator and have then mapped to sensory consequences. Both pattern generators have the same universal form and show autonomous, metastable dynamics of the sort seen in the real brain [87].

**Figure 3:**
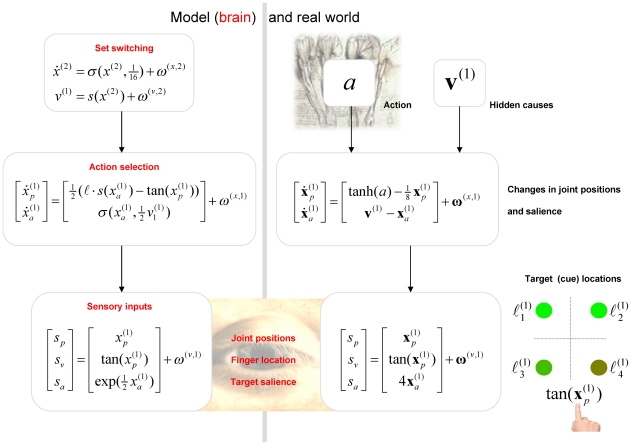
This figure distinguishes between the equations of the generative model (left-hand side; see **Equation 6**) and the equations generating sensory information (right-hand side; see **Equation 5**). The generative model is trying to predict the sensory states produced by the equations on the right. These sensory states comprise the location of the agent's arm in both proprioceptive (intrinsic) and exteroceptive (extrinsic) coordinates. The locations of the four cues in the previous figure are shown in extrinsic coordinates in the lower right insert. In addition to these sensory inputs, the agent also receives sensory information about the salience of cues at the four locations (e.g., illumination). The equations of the generative model have been divided into those responsible for the selection or generation of a particular context or set and those specifying the relative affordance of cue locations used to select action. Crucially, both sets of equations are based on winnerless competition using the itinerant dynamics of the previous figure. These equations come to life when action (driving movements) becomes a function of conditional expectations about hidden variables in the generative model. See main text for further details.

In summary, the agent believes that it will point towards salient cues when they appear. Furthermore, it believes that these cues would appear one at a time; either in a fixed (clockwise) sequence or with no sequential contingencies. Although this is a very simple model of the world, it allows us to demonstrate sensorimotor integration in the context of cued motor actions, biasing of action selection in terms of sequential anticipations and set switching that depends upon recognising the context (sequential or random) in which cues appear. Our particular interest here is in how manipulating the precision (dopamine) at various levels in this hierarchical model will impact on cued responses. The interesting behaviour depends entirely upon the prior beliefs entailed by the form of the generative model and its equations of motion. These are shown schematically in Figure 3, which highlights the difference between the structured and dynamical expectations implicit in the generative model (left panels) and the relatively simple dynamics underlying the generation of sensory input (right panels). This emphasises the fact that real behaviour emerges through the expectations and active sampling of the environment that an agent brings to the world: expectations that are embodied in its generative model. It should be emphasised, that despite the complexity of these models, perception and action can be accounted for by one straightforward principle; namely the minimisation of free energy, as in Equation 1.

**Figure 4:**
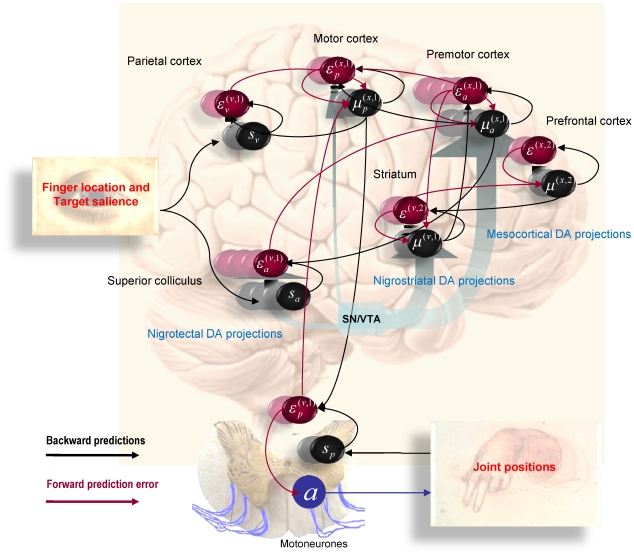
This schematic illustrates the connections between prediction units (black) and error units (red) that underlie the simulated reaching movements. The prediction units encode conditional expectations about hidden states and causes, while the error units encode the associated prediction errors. The connections between these two sorts of units are specified by the message passing scheme in Equation 3 (cf., Figure 1). In brief, error units pass precision weighted prediction errors forward and horizontally (red connections), while prediction units sent predictions backwards and horizontally (black connections). Note that prediction units only communicate with error units and *vice versa*. In this figure, expectations about hidden states in the first level have been divided into two sets, corresponding to the position of the arm (motor cortex) and the affordance of the cue locations (premotor cortex). The blue circle at the bottom of this figure indicates motor neurons in the ventral horn of the spinal cord that mediate action. The cyan arrows represent various projections from the substantia nigra and ventral tegmental area (SN/VTA). Exteroceptive sensory information enters directly at parietal cortex and the superior colliculus encoding positional information about the arm and the salience of cue locations respectively.

If we substitute the generative model in Equation 6 into the message passing (generalised predictive coding) scheme in Equation 3, we arrived at the network architecture shown in Figure 4. To lend this architecture a neurobiological plausibility, we have assigned the prediction and error units to neuronal populations in various cortical and subcortical structures. At the sensory level, we have placed sensory prediction error in extrinsic (visual) coordinates in the parietal cortex and the salience (e.g., illumination) of the four target locations in the superior colliculus; cf., [91]. Predictions about the first level hidden states have been divided into proprioceptive (angular position) and affordance states in the motor and premotor cortex respectively; cf., [92]. The motor cortex sends top-down projections to the parietal cortex and spinal-cord to suppress visual prediction errors and elicit motor reflexes respectively. In contrast, the premotor cortex sends top-down predictions about visual salience to the superior colliculus. Predictions about second level causes and states have been assigned to the basal ganglia and prefrontal cortex respectively. These encode the set (sequential or random context) currently inferred. The basal ganglia and prefrontal cortex exchange predictions and prediction errors through cortico-subcortical loops, while the basal ganglia exchanges signals with the premotor cortex to optimise predictions about affordance. The blue arrows arising from the substantia nigra and ventral tegmental area (SN/VTA) are meant to indicate the main (dopaminergic) projections from this area that we assume modulate the postsynaptic gain of the principal cells (red circles) elaborating prediction errors. The activities of these (nigrotectal, nigrostriatal and mesocortical) dopaminergic projections encode the precision of prediction errors at different levels of the sensorimotor hierarchy. Although the recent literature on the (mesorhombencephalic) nigrotectal pathway, from SN to the superior colliculus, focuses on GABAergic projections, a substantial proportion of nigrotectal projection neurons use dopamine [93], [94], [95].

### Simulations

**Figure 5:**
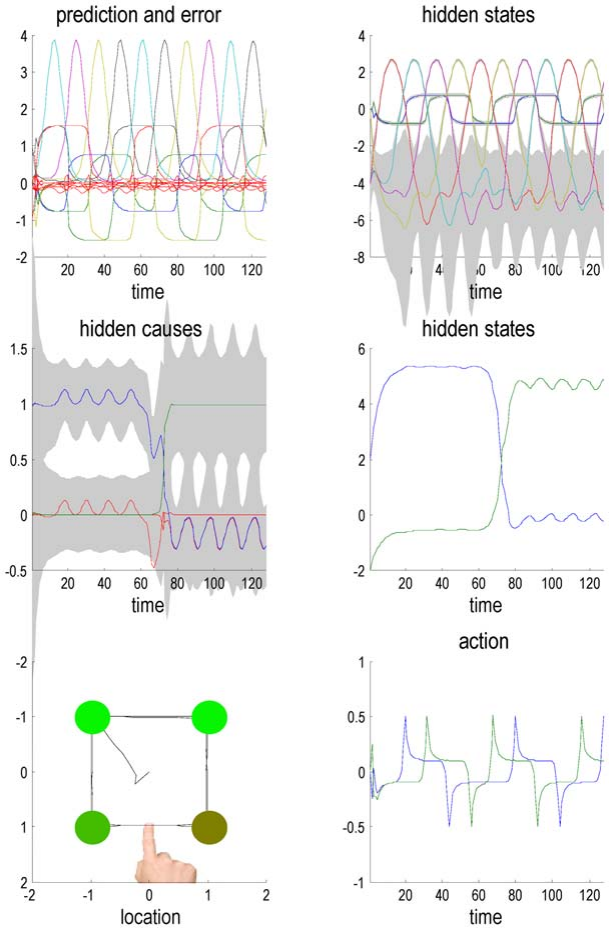
This figure summarizes the results of simulations under normal levels of dopamine (using a log precision of four for all prediction errors). The conditional predictions and expectations are shown as functions of time over 128 time bins, each modeling 64 ms of time. The upper left panel shows the conditional predictions (colored lines) and prediction errors (red lines) based upon the expected in states on the upper right. In this panel and throughout, the grey areas denote 90% Bayesian confidence intervals. The inferred speed of itinerant cycling among affordance states corresponds to the first of the hidden causes at the second level (left middle panel). These hidden causes are a softmax function of their associated hidden states (right middle panel). The blue lines encode a sequential context, while the green lines encode the converse (random) context. The switching in these conditional expectations occurs after sufficient sensory evidence has accumulated following a reversal of the presentation order. The lower left panel shows the trajectory (dotted lines) in an extrinsic frame of reference, in relation to the cue locations (green circles), while the lower right panel shows action in terms of horizontal and vertical angular forces causing these movements.

The model above is sufficient to engender cued reaching movements, which are anticipatory if the agent correctly infers that the cues are presented in a fixed (clockwise) sequence. However, if we reverse the order of the stimuli, there should be accuracy and reaction time costs, due to the fact that the sequence cannot be predicted under clockwise beliefs about the sequence. Furthermore, there should be a set switching cost as the hidden states at the second (context) level are inferred and the itinerant dynamics at the first (affordance) level are suppressed. When we integrated Equation 1, this is precisely what was found:

Figure 5 shows the results of a simulation using log precisions of four (a relatively high precision) throughout the hierarchy. In this example, the target locations appeared every 12 time bins (of 64 ms) using Gaussian bump functions of time. The first five targets were in the (expected) clockwise order, while last five were presented in an anticlockwise order. The resulting conditional predictions and prediction errors are shown in the top four panels of Figure 5, while the trajectory in extrinsic coordinates and the underlying action are shown in the bottom panels (left and right respectively). The upper left panel shows the conditional predictions of sensory signals and sensory prediction errors (in red). These are errors on the salience, proprioceptive and visual input, which, as can be seen, are small in relation to predictions. The predictions were based upon the hidden states shown on the upper right. One can see the itinerant cycling over conditional expectations of hidden affordances (large amplitude lines) that are inferred with a high degree of conditional confidence (the grey areas correspond to 90% Bayesian confidence intervals). The interesting aspect of these results lie in the middle two panels that show the conditional expectations of the hidden causes and states at the second level, encoding the context or set. These results show that it takes about two movements or trials before there is a confident inference that the context has changed. This inferential set switching is driven by the large (downward) deflection in prediction error shown in red (left middle panel). Note that with these precisions, behaviour is accurate and fast and that the violation of sequential expectations is barely discernible. In other words, the precision of sensory information is sufficient to override top-down prior expectations of a sequential sort, when they are clearly violated. However, as we will see later, there is a performance cost in terms of reaction time and accuracy.

**Figure 6:**
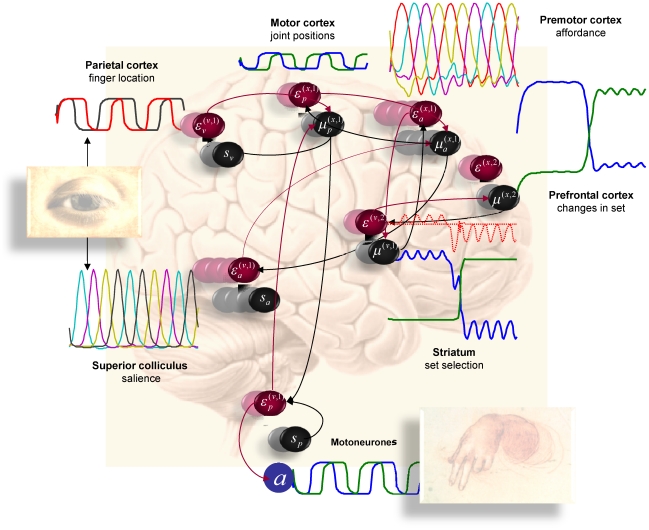
This figure combines the dynamical results from the previous figure with the supposed functional anatomy in **Figure 3**. It shows the conditional expectations about hidden states and causes associated with regionally specific representations. The dotted red time courses associated with the prediction error units in the striatum show a set-related prediction error when the order of the cues was reversed (after the first five presentations). It is these prediction errors that drive the switch in contextual expectations assigned to the prefrontal cortex.

The same simulated neuronal responses are shown in Figure 6, where they are shown alongside their associated brain structure. This figure tries to illustrate how neuronal dynamics unfold at different timescales in different parts of the brain to produce motor behaviour. Crucially, all the hierarchically deployed dynamics are both entrained by and entrain dynamics in lower levels, through the recurrent message passing implicit in generalised predictive coding. By design, we have placed the slower dynamics in higher (more anterior) brain areas [96], [97], [98]–[99], [100]. The neuroanatomical interpretation of this simulation should not be taken too seriously but illustrates the fact that the scheme is (in principle) biologically plausible, both in terms of its dynamical formulation and the functional anatomy of sensorimotor hierarchies in the brain.

### Summary

In summary, we have created a generative model that illustrates the itinerant and dynamic sensorimotor constructs that might be used by the brain to predict cued sequential behaviours and set switching in response to changing contingencies. It is worth noting, that this relatively simple model has implicitly modelled (and integrated) a number of apparently disparate processes in cognitive neuroscience: for example, Bayes-optimal sensorimotor integration, evidence accumulation, anticipation, short term (working) memory, action selection, set switching and a simple form of reversal learning (in terms of switching to a new contingency). We mean this in the straightforward sense that to perform accurately, the simulated agent has to remember the sequence of cues in terms of delay period activity in the premotor and prefrontal cortex [101], encoded here in terms of conditional beliefs about the dynamics of hidden states. Furthermore, to respond optimally the agent has to recognize a reversal in the sequence of cues and adjust its internal representation of context accordingly. Interestingly, [102] presents a model of working memory using exactly the same winnerless (generalized Lotka-Volterra) dynamics used in this paper. Using this model, they show that working memory capacity has an upper bound of seven items, under plausible assumptions about lateral neuronal interactions. Crucially, the cognitive processes like working memory do not need to be modelled explicitly but emerge from the Bayesian inversion of a generative model. In future work, we will use the same model to study learning and working memory; however, our current focus is on how Bayes-optimal behaviour degrades when we reduce the precision of prediction errors:

## Results

### Simulating dopaminergic depletion

In this section, we repeat the above simulations under different levels of precision in the putative targets of dopaminergic projections. This is meant to simulate depleted levels of dopamine; acting at postsynaptic D1 receptors to reduce postsynaptic gain (see Figure 1). First, we reduced the log precision (by 50% in 6 steps) in the principal cells of the superior colliculus that report the prediction errors on the salience of target locations: 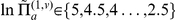.

**Figure 7:**
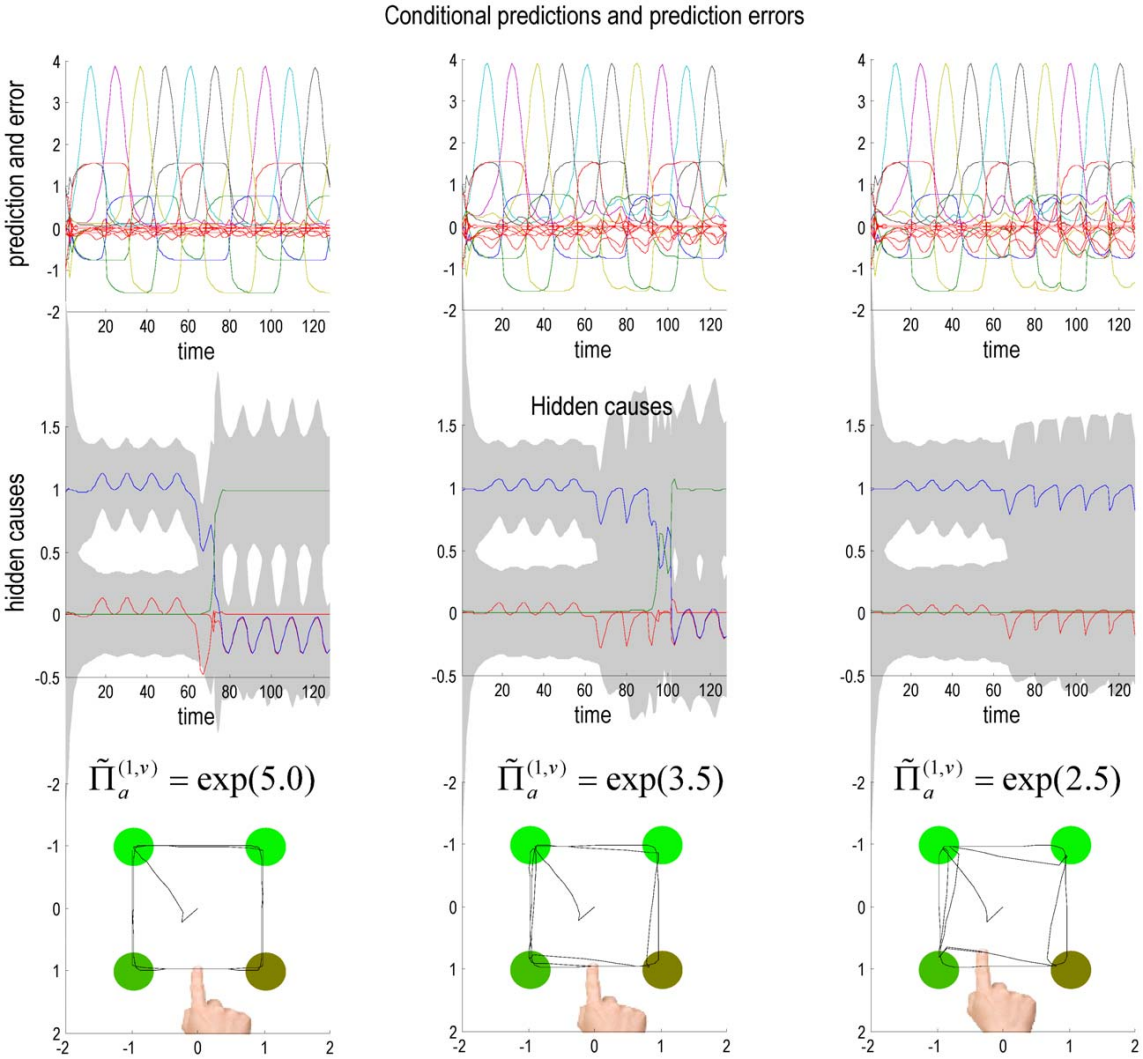
This figure shows the results of simulations under progressively reduced levels of precision (dopamine) as indicated by the equalities in the lower row. The display format of these simulated responses is the same as used in the left panels of Figure 5 (conditional predictions and prediction error; hidden contextual causes at the second level and motor trajectories). The left column presents the conditional responses under normal levels of dopamine (as in Figure 5), while the middle and right columns show the equivalent responses for intermediate and low levels of dopamine. As noted in the main text, the main features of these simulations are reciprocal changes in the amplitude of prediction errors at the first and second levels that are associated with a progressive failure set switching (i.e., a failure to recognise that the order of stimulus presentation no longer conforms to sequential expectations).

The effects on conditional expectations of hidden states and causes are shown in Figure 7 for high, intermediate and low levels of precision (dopamine). The upper row shows the conditional predictions of sensory input and sensory prediction errors as in Figure 5, while the middle row shows the conditional expectations of the hidden causes encoding context. The most remarkable thing about these results is the failure to infer a change in the context (or set) when dopamine is depleted. This results in an accumulation of prediction error at the sensory level while, in contrast, the prediction error at the second level (red lines in the middle panels) decreases. This is an intuitive consequence of decreasing the relative precision at lower levels of the hierarchical model, which causes the inference to be over reliant upon top-down priors and less confident about switching to the new context, when sensory prediction errors are less precise.

**Figure 8:**
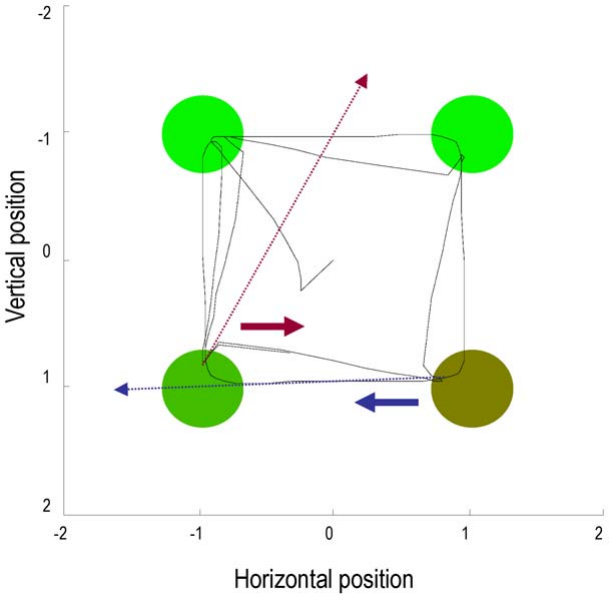
This is a blow up of the motor trajectories under low levels of dopamine from the previous figure. It highlights the fact that when movements are in an expected clockwise direction, the initial trajectory (dotted blue arrow) is directed towards the (correct) next target location. Conversely, when the movements are in a counter clockwise direction, the agent is initially confounded by false expectations about which cue will appear next (dotted red arrow).

This means that it takes longer before the second level expectations accumulate sufficient evidence to make them switch, following the reversal of stimulus order. At the lowest level of simulated dopamine, this switch fails completely and the agent always expects the next target to appear in the wrong (clockwise) location. The behavioural consequences of this are shown graphically in the lower panels of Figure 7, in terms of the trajectory of movements over the ten cues (trials). We see here that the trajectory is perturbed progressively as dopamine levels fall; with initial directions being pulled in the direction of falsely anticipated target locations. This is shown more clearly in Figure 8, which shows the trajectory for the lowest level of dopamine. During the first five trials the initial excursion from the lower right target is in the correct direction for the next target in the (clockwise) sequence. However, after the reversal, the initial trajectory from the lower left target is drawn towards the incorrectly anticipated next target, requiring a corrective adjustment to the movement trajectory, when the actual target discloses itself.

**Figure 9:**
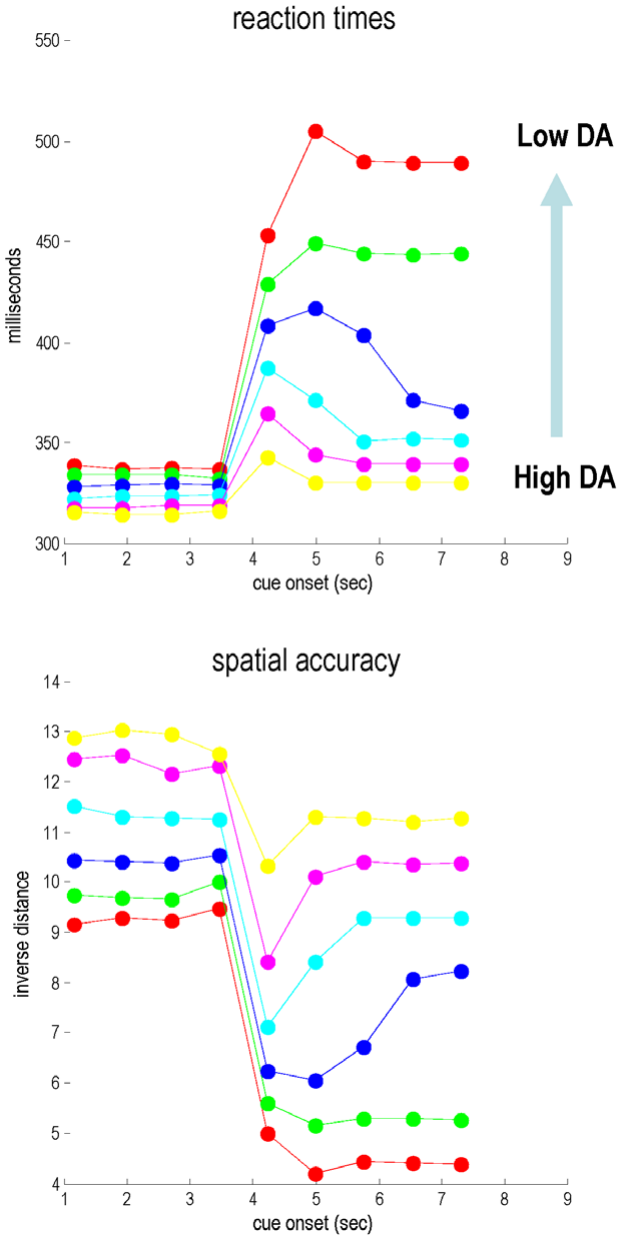
This figure presents the behavioral results from the simulations under different levels of dopamine. The upper panel shows the reaction times for each trial or cue as a function of cue order (over 10 cues). The reaction time was measured as the time from cue onset to the time that the pointing location fell within a small distance of the target location. The equivalent results for accuracy are shown in the lower panel in terms of the (inverse) average distance from the pointing location to the target location for each trial. The colored lines correspond to different levels of simulated dopamine; with red lines indicating the lowest level and yellow lines the highest. The key things to note here are: (i) the reaction time costs of unpredictable (first five), relative to predictable trials (first five), shown by the yellow line and (ii) the increase in amplitude and duration of switching costs as dopamine is depleted (colored lines); modeled here in terms of the precision of prediction errors on visual salience.

Figure 9 shows the behavioural consequences of this precision or dopamine-dependent failure to correctly infer the sequential context: the top panel shows the reaction times (assuming 64 ms time bins) measured as the time from the onset of the cue to the time at which the target was reached (to within a radius of 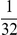). The corresponding spatial accuracy is shown in the lower panel as a weighted average of the (inverse) distance to target during each trial. There are two important things to take from these results:

First, irrespective of the level of dopamine, reaction times are faster when the next cue can be anticipated. Furthermore, there is a price to be paid for this anticipatory speeding, when sequential anticipations are violated. This is reflected in the increased reaction times at the point of sequence reversal for a couple of trials. It is these transient decreases in performance that index the switching costs hypothesised earlier. Crucially, the effect of dopamine depletion is to exacerbate both the switching costs and the behavioural slowing when sequential predictions no longer hold. In the limit of very low dopamine, and a complete failure to switch sets (infer a context change), there is a marked impairment in performance that persists following reversal. Perhaps the most important result in Figure 9 is that the set switching costs persist for longer with low levels of dopamine. In other words, there is a perseveration of (suboptimal) anticipatory motor trajectories that is exacerbated by dopamine depletion. This latent bradykinesia and perseveration is reminiscent of the symptoms of Parkinson's disease [103], [104], which was the motivation for these simulations.

On the basis of these results, one might predict that the greatest difference between Parkinson's patients on and off dopaminergic medication would be expressed most acutely in trials that violated expectations established during sequential cueing. Conversely, there should be relatively small differences in reaction times when stimuli are presented in the correct sequence: cf., [105]. Furthermore, differences in reaction times with unpredictable cues should not be marked, once patients have realised that there is no underlying sequence. We will consider these predictions in relation to empirical results in a forthcoming paper. It is also interesting to relate these simulations to the results in [106], who found deficits in probabilistic reversal learning in Parkinson's disease, where “patients also exhibited compromised adaptability to the reversal”. Brown and Marsden [107] investigated set switching in Parkinson's disease using the Stroop task. Subjects had to report either the semantic or physical colour of a word; however, the rule changed every ten trials. The response dimension was cued before each trial or subjects were just reminded to change the rule every ten trials. Patients showed general psychomotor slowing but were further impaired on the uncued condition, especially in the first trial following a rule change.

**Figure 10:**
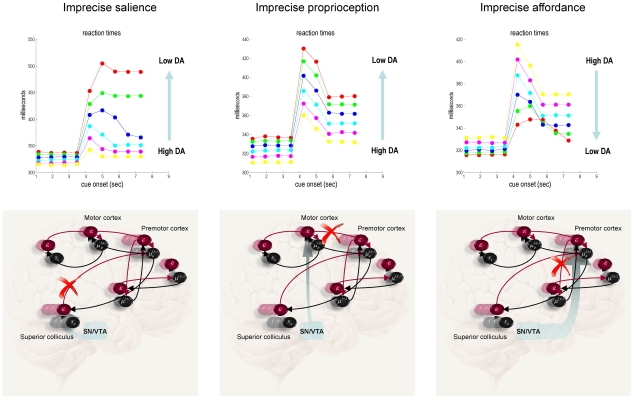
This figure represents behavioral results in terms of reaction times for depleting dopamine in three regions: the superior colliculus encoding sensory salience (as in previous figure), the motor cortex encoding proprioception (middle column) and the premotor cortex encoding affordance (right column). These results are shown using the same format as in previous figure and illustrate the qualitatively different effects of dopamine depletion in different parts of the brain (or model). The lower panels indicate the implicit projections, from the substantia nigra or ventral tegmental area, have been selectively depleted (where a red cross highlights the forward prediction errors affected). The key thing to take from these simulations is that reducing the precision of prediction errors on sensory salience induces bradykinesia and perseveration; whereas the equivalent reduction in proprioceptive affordance causes bradykinesia without perseveration. Finally, compromising the precision of changes in affordance increases perseveration and decreases bradykinesia.

Finally, we repeated the simulated lesion experiments above by reducing the precision in other cortical and subcortical structures in receipt of SN/VTA projections. The most interesting results are shown in Figure 10 in terms of simulated reaction times over different levels of dopamine: the left panels reproduce the reaction time data of the previous figure, while the middle panel shows the equivalent results obtained when depleting dopamine in the motor cortex (encoding conditional expectations about proprioceptive inputs). The effect of dopamine depletion here is to increase reaction times in a non-specific way. This non-specific slowing was expected, as proprioceptive prediction errors are subverted thereby reducing motor vigour; cf., [108]. Note that inference about affordance and set are not affected, because these are driven by exteroceptive prediction errors. This means there is no change in set switching or perseveration. Conversely, when we lesion mesocortical projections to the premotor cortex (modulating prediction errors about changes in affordance) there are effects on both motor vigour and set switching (right hand panels). Importantly, these effects differ from those produced by the same lesion to the superior colliculus. Here, depleting dopamine actually *decreases* reaction times. This is perfectly sensible because increasing the precision at higher levels of a hierarchical model has the opposite effect to increasing the precision of lower level prediction errors. In other words, as dopamine levels in the premotor cortex increase, the agent becomes overly confident about its top-down (empirical) prior expectations, even in the face of precise sensory information. This overconfidence is manifest in terms of a slight impairment in reactions elicited by sensory cues. However, the overconfidence about affordance enables the second level of the generative model to recognise the change in context more efficiently (quickly). This induces a trade-off between the efficiency with which cues elicit movements and the efficiency of set switching.

### Summary

In this section, we have seen how simulated dopamine depletion is manifest in terms of neuronal responses encoding Bayes-optimal inferences about sensorimotor contingencies and in terms of behavior. The key point made by the simulations is that although dopamine may have a singular mechanism of action and computational function (e.g., to modulate postsynaptic gain and encode precision) the physiological and behavioral correlates of dopamine changes depend on where in the brain they are expressed. This reiterates the point made in the introduction that understanding the role of dopamine may call for a multilateral perspective that accommodates the delicate balance among distributed responses that underlie the functional anatomy of behavior. The simulations in this section can be regarded as a proof of principle that a single mechanism can lead to the diverse functional consequences seen empirically [109], [7], [110]. Here, dopamine had opposite effects on the speed of movements (bradykinesia) depending upon whether it was depleted at higher or lower levels of the sensorimotor hierarchy. Conversely, simulated depletion of dopamine in the superior colliculus (low-level) and premotor cortex (high-level) had similar effects on perseveration. We emphasize this point because it has implications for the computational modeling of dopamine, especially for theoretical accounts of dopamine that consider one optimization process in isolation (for example, associating dopamine with reward prediction error). A nice example of the plurality of deficits following insults to be dopamine system is provided by [111], who conclude that Parkinson's “patients on and off medication both showed attentional shifting deficits, but for different reasons. Deficits in non-medicated patients were consistent with an inability to update the new attentional set, whereas those in medicated patients were evident when having to ignore distractors that had previously been task relevant.”

In summary, contrary to what is often assumed, dopamine may not report the prediction error on value but the value (precision) of prediction errors. If this is the case, one would anticipate different behavioral deficits following dopamine depletion, depending on which prediction errors were affected. Strategically, it may be better to ask not what the function of dopamine tells you about a model but what a model tells you about the function of dopamine. In this paper, the function of dopamine is to modulate postsynaptic gain, while the model mediates between this (neuronal) function and its behavioral and neurophysiologic consequences. See also [11].

## Discussion

In this paper, we have presented a simple model of cued reaching movements and set switching that is consistent with the notions of salience and affordance. Furthermore, we have simulated some latent symptoms of Parkinsonism by reducing the precision of cues that have affordance. Reducing this precision (dopamine) delays and can even preclude set switching, with associated costs in behavioral accuracy. When the precision of sensory cues is removed completely, we obtain autonomous behavior that is prescribed by the itinerant expectations of the agent (results not shown). Crucially, these simulations are not based on an *ad hoc* model of dopaminergic function but use exactly the same principles, equations and numerics used previously to address a wide variety of processes in cognitive neuroscience: Table 1 lists the growing number of paradigms and processes that can be explained in terms of free energy minimization and hierarchical Bayesian inference. The simulations in this and related papers can be reproduced from a graphical user interface available in Matlab code (http://www.fil.ion.ucl.ac.uk/spm/).

In short, we have focused on the role of dopamine in balancing the influence of bottom-up sensory information and top-down (empirical) prior expectations during perceptual inference and consequent behavior. This role is consistent with many other theoretical treatments of dopamine in modulating top-down effects and signal-to-noise [49], [44], [11], [112], [2], [113], [45], [39], [40]; and contextualizes them within the formal setting of generalized predictive coding.

### Functional anatomy and dopamine

The simulations presented in this paper do not attempt to cover all the physiological and computational processes that dopamine may mediate. Our results pertain to classical neuromodulatory postsynaptic consequences of changes in tonic dopamine release (e.g., during depletion due to drugs or disease) on principal cells. There are many aspects of dopaminergic function that are not addressed by these simulations: for example, modulation of long-term synaptic plasticity, or the differential roles of various pre- and postsynaptic dopamine receptors. Furthermore, we have only considered the effect of tonic or baseline dopamine levels (over long time scales) and have ignored dopamine fluctuations on a shorter time scale: for example, the phasic release of dopamine in response to specific cues or its rapid clearance in the striatum by the dopamine active transporter. However, the aim of the present work was to highlight the computational, neurophysiologic and anatomic considerations that suggest a unitary role for dopamine in the encoding of precision.

There is a large literature on modeling dopamine in the context of choice and motor behavior [114], [115], [116], [117], [118], [119]. Some of this literature is based on reinforcement learning and optimal decision theory; and formulated at a rather abstract level in terms of discrete state spaces and time. It is difficult to connect the (predictive coding) process model provided in this paper with the more descriptive (but useful) heuristics provided by reinforcement learning and economics. One link may be via variational Bayesian formulations of reinforcement-learning update equations (see below), in which precision-weighted prediction errors play a key role [120]. On the other hand, there are computational simulations whose biological detail goes beyond the simulations in this paper [112], [121], [122], [123]. In this sense, the simulations reported here should be regarded as an illustration of how far one can get in modeling neuromodulation in the larger setting of Bayes optimal action and perception. In this context, *Bayes optimality* (implicit in free energy minimization) replaces *optimal control* (implicit in reinforcement or value learning).

The ideas and simulations in this paper just show that dopamine fits comfortably into a principled if somewhat abstract formulation of action and perception. Specifically, it has the physiological characteristics that would be required to fulfill a central role in Bayes-optimal inference; namely, to encode precision or uncertainty. This is why we have focused on the neuromodulation of postsynaptic gain in cortical cells, mediated by D1 receptors. The theoretical constraints we have considered do not really allow us to say very much about the functional anatomy of the cortico-basal ganglia-thalamic loops. Having said this, the dynamics necessary for active inference are not inconsistent with many aspects of known functional anatomy. For example, the basal ganglia are believed to act on cortex through a process of focused disinhibition [124]. In particular, the basal ganglia output nuclei fire tonically to inhibit cortical activity, but strong striatal activity can reduce this tonic activity to release target thalamic areas and their corresponding cortical areas from inhibition. This basic functional architecture is reflected in our simulations by the selection of an appropriate sensorimotor set by representations of hidden causes in the striatum that selectively enable particular attractors or central pattern generators in premotor cortex. Note that focused disinhibition is a necessary consequence of the dynamics on hidden states and causes that mediate winnerless competition. In one sense, it is difficult to imagine any alternative sort of dynamics that could prescribe unique sequential behavior and motor trajectories.

Although the computations that minimize free energy do not predict the details of functional anatomy (in the same way that maximizing adaptive fitness does not predict the details of a phenotype), it does provide some interesting insights into large-scale functional anatomy. These insights rest upon the notion that the brain is a generative model of its environment. This means that causal regularities in the environment should be recapitulated in its neuroanatomy. The trick here is to think of the brain as generating predictions of sensory input using its backward (top-down) connections; in other words, to imagine the brain without the forward connections that are used for perceptual inversion of its generative model. The picture that emerges is a fine lacework of cortical projections from high-order associative cortex that radiate to sensory cortical areas and then send top-down predictions of sensory input to thalamic nuclei. A nice example here is the distinction between what and where pathways in visual processing [125]. Their very existence suggests that the (hidden) causes of visual inputs to the lateral geniculate nucleus are objects that can belong to different categories and, crucially, can be in different places. One might imagine that in a static universe, where objects were bound to a particular place, the brain would not have separate representations of what and where or their neuroanatomical correlates. So what does this sort of analysis imply for the basal ganglia? We will focus on three specific predictions:

First, the fact that the outputs of the basal ganglia are to thalamic nuclei suggests that the basal ganglia are at a hierarchically lower level than prefrontal cortex. For example, the ventral lateral nucleus receives inputs from the basal ganglia (via the thalamic fasciculus) and sends outputs to the primary motor cortex. The ventral lateral nucleus is juxtaposed to the ventral posterolateral nucleus in receipt of proprioceptive and somatosensory afferents via the posterior column-medial lemniscus pathway. The implicit hierarchical level of the basal ganglia suggests that the prefrontal cortical projections to the striatum should be of the backwards type (originating from deep pyramidal cells). Indeed, the cortical pyramidal neurons projecting to the striatum are located in layers II–VI, but the densest projections come from layer V [126]. Furthermore, the medium spiny neurons that receive these corticostriatal projections express dopaminergic (D1 - direct and D2 - indirect pathway) receptors [127]; as would be expected, if they played the role of prediction error units that are targeted by backward connections.

Second, in active inference, the balance between top-down prior beliefs and bottom-up sensory information is controlled by the relative precision of prediction errors at higher and lower levels of cortico-basal ganglia-thalamic hierarchy. This suggests that there will be an antagonistic control of dopaminergic projections to the prefrontal cortex (higher level) and equivalent projections to the striatal (lower-level). In anatomical terms, this may be reflected in the segregation of the cells of origin of the nigrostriatal dopamine projections (from the substantia nigra) and the cortical projections (from the ventral tegmental area). Clearly, there are several important functional associations in dopaminergic systems that may or may not be understandable at this level of analysis. Key examples here include the opposing effects of the direct and indirect pathways [128], [129] and the opposing influences of dopamine on synaptic plasticity [56].

Finally, it is interesting to note that the number of parameters of statistical models is generally much greater than the degrees of freedom of their variance or precision estimates. For example, a classical statistical model with both between and within subject factors can have hundreds of parameters yet can be inverted using just two precisions (modeling within and between subject variability). This might be a simple explanation for the fact that the number of dopaminergic cells (encoding precision or context) is much smaller than the myriad of cells (encoding hidden causes or content) needed to parameterize the world.

### Prior beliefs or rewards?

The current work presents a covert challenge to standard theories of decision-making and motor control based upon the concept of reward or value. As hinted at in the introduction, there is an inherent circularity in reward-based accounts of behavior that may obscure deeper questions about behavior. This is because (psychological) reward is defined as a process that reinforces or modifies behavior. As such, it is circular to use reward to explain behavior. Although the concept of reward may provide beautifully self-consistent descriptions of behavior in terms of optimizing expected reward; e.g., [20], reward-based accounts (and their neurophysiologic correlates) cannot explain behavior *per se*. The alternative and simpler approach, provided by active inference, replaces rewards with prior beliefs about how the world should unfold and motivates these priors in evolutionary and ethological terms. Put simply, survival does not depend on seeking out rewards; it depends upon avoiding surprising encounters and physiological states that are uncharacteristic of a given phenotype. It is therefore sufficient to minimize surprise (free energy), which is a problem of (active) inference, not reinforcement learning.

At no point in the formulation above do we refer to reward or value; the only optimization was to minimize surprise about expected outcomes. In the context of hierarchical inference, the potentially potent role of dopamine is obvious. This is because the delicate mixing of bottom-up sensory information, with top-down priors, to produce conditional beliefs (and action) is exquisitely sensitive to the precision or certainty ascribed to representations at different levels of a hierarchy. This is a generic and ubiquitous aspect of hierarchical inference; for example, inference about treatment effects in group studies rests on veridical estimates of within-subject, relative to between-subject variability (precision). The idea here is that dopamine reports the precision (variability) in hierarchical models used by the brain to infer the causes of sensory data. In this view, the task instructions and cues provided to subjects in decision-making experiments do not, in themselves, constitute rewards but are used to instill prior beliefs about how they should behave.

Although we have questioned the epistemological status of reward on the grounds of circularity, one cannot deny that certain cues are inherently rewarding; for example, those induced by appetitive stimuli. In this sense, one might associate reward with the affordance of (hidden) causes in the environment that elicit obligatory volitional and autonomic responses. In this context, the tautology of reward is resolved by noting that reward is a perceptual attribute of a cue not a cause of behavior: in active inference, behavior is caused by prior beliefs and ensuing exchange with environment. These beliefs ensure certain states are occupied frequently, where these states are, by definition, valuable. In this view, value and reward are consequences not causes of behavior. Behavior is caused by the environment and prior beliefs that are established epigenetically or through experience dependent learning. This means that reward is a perceptual (hedonic) consequence of behavior, not a cause.

The argument in this paper is that the information (prediction error) conveyed by rewarding cues cannot be encoded by dopamine, because dopamine cannot excite postsynaptic the responses that would be needed to mediate the influence of that information: Dopamine can only modulate the postsynaptic responses to glutamatergic or other neurotransmitter release. This is not to say that rewarding cues will not excite dopamine cells. Indeed, this is the hypothesis put forward here – namely that rewards or cues with particular affordance can select exteroceptive, interoceptive and proprioceptive processing channels that mediate behavioral responses. In short, dopamine may report the precision or confidence about reward, not reward *per se*. This account may explain why dopaminergic responses do not behave as reward prediction errors generally. For example, a significant proportion of dopamine neurons increase their firing to aversive stimuli, and cues which predict them [130], [131]. This is the opposite behavior to that predicted by the reward prediction error hypothesis, but follows naturally from the precision encoding framework, since both aversive and appetitive cues signal predictable sensorimotor sequelae. The precision hypothesis also accounts for the finding that dopaminergic neurons fire in response to stimuli that predict the subsequent availability of predictive information about upcoming reward, even though such stimuli convey no information about the reward itself [132]. On the account presented in this paper, such stimuli elicit dopaminergic activity because they signal the onset of a predictable sequence of events. Accounting for all observed patterns of dopaminergic activity falls outside the scope of this paper, but it is clear that the precision hypothesis predicts the involvement of dopamine in a broader range of processes than some existing accounts.

The simulations in this paper focused on the depletion of dopamine. However, increasing levels of, or sensitivity to, dopamine in the basal ganglia has been implicated in several movement disorders, including tardive dyskinesia [133], Huntington's disease [134], Tourette syndrome [135], hemiballismus [136] and levodopa-induced dyskinesia [137]. These disorders produce involuntary, non-purposeful movements that are nonetheless more structured than simple myoclonic jerks or tremor. If dopaminergic transmission was increased pathologically in lower levels of the hierarchy, fluctuations in ascending (afferent) prediction errors could become sufficiently precise (potent) to trigger movement; in other words, subliminal cues would be awarded aberrant affordance and elicit inappropriate action.

### Precision and learning rates

Although we have not dealt with the phasic release of dopamine and the implicit optimization of precision, the current formulation does make some strong predictions about the modulation of stimulus bound or event related responses. If tonic dopaminergic levels modulate postsynaptic gain, one would expect to see amplified responses in the post synaptic targets of dopamine projections that report (sensory) prediction errors. This means that one might expect greater evoked responses to the same stimuli when presented in a predictable, as opposed to an unpredictable context. Indeed, this is the basis of an explanation for the mismatch negativity: an increase in the event related potential elicited by a novel or oddball stimulus, presented in a predictable train of stimuli. The proposed mechanism rests on prediction errors that are afforded too much precision [138].

The central role of precision-weighted prediction errors also emerges from other computational perspectives. For example, one can formulate dynamic inference about the causes of sensory cues without reference to neurobiology but as a generic hierarchical Bayesian model [120]. Variational Bayesian methods then yield the same sort of precision-weighted prediction errors as in the present work. Crucially, the variational update equations are formally related to reinforcement learning rules; where precisions play the role of learning rates. This means that one could test whether dopaminergic drug manipulations affect estimates of precision (learning rates) based on empirical behavioral responses during classical reinforcement learning paradigms.

We have not touched upon learning *per se*, because we have assumed that the prior beliefs used to generate behavior were already known in the simulations. In future work, we will use the simulations above to look at the acquisition of prior beliefs in terms of sequence learning and the effect that dopamine has on this learning. The key issue here is that any effects of dopamine on learning and activity-dependent plasticity (using free energy minimization) are mediated vicariously through its effects on neuronal activity. This means that the only thing specific about dopamine, in the context of learning, is due to the restriction of its projections to the (extended) motor system. This suggests that sensory learning will be largely unaffected by dopaminergic manipulations (simulated or real) to the extent that it is independent of motor learning. We will explore this in future work, in which we will treat (phasic) dopamine release as encoding state-dependent precision, given contextual cues like conditioned stimuli: see [132]. Formally, this is closely related to the use of state-dependent precision to understand attentional gain in perception [30].

One interesting aspect of the simulations in this paper is that they lend themselves nicely to higher-order (operant) learning paradigms: The behavior generated by high-level sensorimotor constructs does not distinguish between sensations caused by motor acts or *vice versa*, because distinct exteroceptive and motor predictions only arise at lower (sensory) levels of the hierarchy. In other words, we could regard the simulations above as simulations of actions that disclose salient cues (as opposed to salient cues causing action). This perspective may be useful when trying to understand the central role of dopamine in action selection based upon conditioned stimuli on the one hand, and optimizing behavior to access conditioned reinforcers on the other.

## References

[1] Friston KJ, Daunizeau J, Kiebel SJ (2009). Active inference or reinforcement learning?. PLoS One.

[2] Cisek P (2007). Cortical mechanisms of action selection: the affordance competition hypothesis.. Philos Trans R Soc Lond B Biol Sci.

[3] Gibson JJ, R S, Bransford J (1977). The theory of affordances.. Perceiving, acting, and knowing: Toward an ecological psychology.

[4] Gibson JJ (1979). The ecological approach to visual perception.

[5] Nitsche MA, Monte-Silva K, Kuo MF, Paulus W (2010). Dopaminergic impact on cortical excitability in humans.. Rev Neurosci.

[6] Maia TV, Frank MJ (2011). From reinforcement learning models to psychiatric and neurological disorders.. Nat Neurosci.

[7] Cools R, Barker RA, Sahakian BJ, Robbins TW (2001). Enhanced or impaired cognitive function in Parkinson's disease as a function of dopaminergic medication and task demands.. Cereb Cortex.

[8] Wiecki TV, Frank MJ (2010). Neurocomputational models of motor and cognitive deficits in Parkinson's disease.. Prog Brain Res.

[9] van Swinderen B, Andretic R (2011). Dopamine in Drosophila: setting arousal thresholds in a miniature brain.. Proc Biol Sci.

[10] Braver TS, Barch DM, Cohen JD (1999). Cognition and control in schizophrenia: a computational model of dopamine and prefrontal function.. Biol Psychiatry.

[11] Hazy TE, Frank MJ, O'Reilly RC (2006). Banishing the homunculus: making working memory work.. Neuroscience.

[12] Dayan P (2009). Dopamine, reinforcement learning, and addiction.. Pharmacopsychiatry.

[13] Redish AD (2004). Addiction as a computational process gone awry.. Science.

[14] Braver TS, Barch DM (2002). A theory of cognitive control, aging cognition, and neuromodulation.. Neurosci Biobehav Rev.

[15] McClure SM, Daw ND, Montague PR (2003). A computational substrate for incentive salience.. Trends Neurosci.

[16] Berridge KC (2007). The debate over dopamine's role in reward: the case for incentive salience.. Psychopharmacology (Berl.).

[17] Kakade S, Dayan P (2002). Dopamine: generalization and bonuses.. Neural Netw.

[18] Schultz W, Dayan P, Montague PR (1997). A neural substrate of prediction and reward.. Science.

[19] Joel D, Niv Y, Ruppin E (2002). Actor-critic models of the basal ganglia: new anatomical and computational perspectives.. Neural Netw.

[20] Montague PR, Hyman SE, Cohen JD (2004). Computational roles for dopamine in behavioural control.. Nature.

[21] Zhang J, Berridge KC, Tindell AJ, Smith KS, Aldridge JW (2009). A neural computational model of incentive salience.. PLoS Comput Biol.

[22] Friston KJ, Daunizeau J, Kilner J, Kiebel SJ (2010). Action and behavior: a free-energy formulation.. Biol Cybern.

[23] Friston K, Mattout J, Kilner J (2011). Action understanding and active inference.. Biol Cybern.

[24] Skinner BF (1938). The Behavior of Organisms. An Experimental Analysis.

[25] Bellman R (1952). On the Theory of Dynamic Programming.. Proc Natl Acad Sci U S A.

[26] Sutton RS, Barto AG (1981). Toward a modern theory of adaptive networks: expectation and prediction.. Psychol Rev.

[27] Diedrichsen J, Shadmehr R, Ivry RB (2010). The coordination of movement: optimal feedback control and beyond.. Trends Cogn Sci.

[28] Friston K (2005). A theory of cortical responses.. Philos Trans R Soc Lond B Biol Sci.

[29] Redgrave P, Gurney K (2006). The short-latency dopamine signal: a role in discovering novel actions?. Nat Rev Neurosci.

[30] Feldman H, Friston KJ (2010). Attention, uncertainty, and free-energy.. Front Hum Neurosci.

[31] Allport DA, Heuer H, Sanders AF (1987). Selection for action: Some behavioral and neurophysiological considerations of attention and action.. Perspectives on perception and action.

[32] Goldberg ME, Segraves MA (1987). Visuospatial and motor attention in the monkey.. Neuropsychologia.

[33] Deubel H, Schneider WX (1996). Saccade target selection and object recognition: evidence for a common attentional mechanism.. Vision Res.

[34] Bestmann SHLM, Blankenburg F, Mars R, Haggard P, Friston KJ (2008). Influence of uncertainty and surprise on human corticospinal excitability during preparation for action.. Curr Biol.

[35] Baldauf D, Deubel H (2010). Attentional landscapes in reaching and grasping.. Vision Res.

[36] Dalrymple KA, Kingstone A (2010). Time to act and attend to the real mechanisms of action and attention.. Br J Psychol.

[37] Gherri E, Eimer M (2010). Manual response preparation disrupts spatial attention: an electrophysiological investigation of links between action and attention.. Neuropsychologia.

[38] Hersch SM, Ciliax BJ, Gutekunst CA, Rees H, Heilman CJ (1991). Electron microscopic analysis of D1 and D2 dopamine receptor proteins in the dorsal striatum and their synaptic relationships with motor corticostriatal afferents.. J Neurosci.

[39] Cisek P, Kalaska JF (2010). Neural mechanisms for interacting with a world full of action choices.. Annu Rev Neurosci.

[40] Anselme P (2010). The uncertainty processing theory of motivation.. Behav Brain Res.

[41] Doya K (2008). Modulators of decision making.. Nat Neurosci.

[42] Rushworth MF, Behrens TE (2008). Choice, uncertainty and value in prefrontal and cingulate cortex.. Nat Neurosci.

[43] Schultz W (2007). Multiple dopamine functions at different time courses.. Annu Rev Neurosci.

[44] Fiorillo CD, Tobler PN, Schultz W (2003). Discrete coding of reward probability and uncertainty by dopamine neurons.. Science.

[45] Rolls ET, Loh M, Deco G, Winterer G (2008). Computational models of schizophrenia and dopamine modulation in the prefrontal cortex.. Nat Rev Neurosci.

[46] Winterer G, Weinberger DR (2004). Genes, dopamine and cortical signal-to-noise ratio in schizophrenia.. Trends Neurosci.

[47] Berridge KC, Robinson TE (1998). What is the role of dopamine in reward: hedonic impact, reward learning, or incentive salience?. Brain Res Brain Res Rev.

[48] Kapur S (2003). Psychosis as a state of aberrant salience: a framework linking biology, phenomenology, and pharmacology in schizophrenia.. Am J Psychiatry.

[49] Gurney K, Prescott TJ, Redgrave P (2001). A computational model of action selection in the basal ganglia. I. A new functional anatomy.. Biol Cybern.

[50] Ashby FG, Casale MB (2003). A model of dopamine modulated cortical activation.. Neural Netw.

[51] Frank MJ (2005). Dynamic dopamine modulation in the basal ganglia: a neurocomputational account of cognitive deficits in medicated and nonmedicated Parkinsonism.. J Cogn Neurosci.

[52] Moustafa AA, Gluck MA (2011). A neurocomputational model of dopamine and prefrontal-striatal interactions during multicue category learning by Parkinson patients.. J Cogn Neurosci.

[53] Schultz W, Preuschoff K, Camerer C, Hsu M, Fiorillo CD (2008). Explicit neural signals reflecting reward uncertainty.. Philos Trans R Soc Lond B Biol Sci.

[54] Plotkin JL, Day M, Surmeier DJ (2011). Synaptically driven state transitions in distal dendrites of striatal spiny neurons.. Nat Neurosci.

[55] Vickery TJ, Chun MM, Lee D (2011). Ubiquity and Specificity of Reinforcement Signals throughout the Human Brain.. Neuron.

[56] Shen W, Flajolet M, Greengard P, Surmeier DJ (2008). Dichotomous Dopaminergic Control of Striatal Synaptic Plasticity.. Science.

[57] Friston K, Kilner J, Harrison L (2006). A free energy principle for the brain.. J Physiol Paris.

[58] Gregory RL (1980). Perceptions as hypotheses.. Phil Trans R Soc Lond B.

[59] Dayan P, Hinton GE, Neal R (1995). The Helmholtz machine.. Neural Comput.

[60] Knill DC, Pouget A (2004). The Bayesian brain: the role of uncertainty in neural coding and computation.. Trends Neurosci.

[61] Yuille A, Kersten D (2006). Vision as Bayesian inference: analysis by synthesis?. Trends Cogn Sci.

[62] Monchi O, Petrides M, Doyon J, Postuma RB, Worsley K (2001). Neural Bases of Set-Shifting Deficits in Parkinson's Disease.. J Neurosci.

[63] Rutledge RB, Lazzario SC, Lau B, Myers CE, Gluck MA (2009). Dopaminergic drugs modulate learning rates and perseveration in Parkinson's patients in a dynamic foraging task.. J Neurosci.

[64] Ginzburg VL, Landau LD (1950). On the theory of superconductivity.. Zh Eksp Teor Fiz.

[65] Haken H (1983). Synergetics: An introduction. Non-equilibrium phase transition and self-selforganisation in physics, chemistry and biology. 3rd edition.

[66] Friston K (2009). The free-energy principle: a rough guide to the brain?. Trends Cogn Sci.

[67] Friston K, Stephan K, Li B, Daunizeau J (2010). Generalised Filtering.. Math Probl Eng vol..

[68] Rao RP, Ballard DH (1999). Predictive coding in the visual cortex: a functional interpretation of some extra-classical receptive-field effects.. Nat Neurosci.

[69] Friston K (2008). Hierarchical models in the brain.. PLoS Comput Biol.

[70] Friston K, Kiebel S (2009). Cortical circuits for perceptual inference.. Neural Netw.

[71] Mumford D (1992). On the computational architecture of the neocortex. II.. Biol Cybern.

[72] Missale C, Nash SR, Robinson SW, Jaber M, Caron MG (1998). Dopamine receptors: from structure to function.. Physiol Rev.

[73] D'Souza UM, Neve K (2009). Gene and Promoter Structures of the Dopamine Receptors.. Dopamine Receptors.

[74] Smiley JF, Levey AI, Ciliax BJ, Goldman-Rakic PS (1994). D1 dopamine receptor immunoreactivity in human and monkey cerebral cortex: predominant and extrasynaptic localization in dendritic spines.. Proc Natl Acad Sci U S A.

[75] Bergson C, Mrzljak L, Smiley JF, Pappy M, Levenson R (1995). Regional, cellular, and subcellular variations in the distribution of D1 and D5 dopamine receptors in primate brain.. J Neurosci.

[76] Yao WD, Spealman RD, Zhang J (2008). Dopaminergic signaling in dendritic spines.. Biochem Pharmacol.

[77] Krimer LS, Jakab RL, Goldman-Rakic PS (1997). Quantitative three-dimensional analysis of the catecholaminergic innervation of identified neurons in the macaque prefrontal cortex.. J Neurosci.

[78] Goldman-Rakic PS, Lidow MS, Smiley JF, Williams M (1992). The anatomy of dopamine in monkey and human prefrontal cortex.. J Neural Transm.

[79] Lidow MS (1995). D1- and D2 dopaminergic receptors in the developing cerebral cortex of macaque monkey: a film autoradiographic study.. Neuroscience.

[80] Davidoff SA, Benes FM (1998). High-resolution scatchard analysis shows D1 receptor binding on pyramidal and nonpyramidal neurons.. Synapse.

[81] Lewis DA, Melchitzky DS, Sesack SR, Whitehead RE, Auh S (2001). Dopamine transporter immunoreactivity in monkey cerebral cortex: regional, laminar, and ultrastructural localization.. J Comp Neurol.

[82] Berger B, Gaspar P, Verney C (1991). Dopaminergic innervation of the cerebral cortex: unexpected differences between rodents and primates.. Trends Neurosci.

[83] Lidow MS, Goldman-Rakic PS, Gallager DW, Rakic P (1991). Distribution of dopaminergic receptors in the primate cerebral cortex: quantitative autoradiographic analysis using [3H]raclopride, [3H]spiperone and [3H]SCH23390.. Neuroscience.

[84] Kubota Y, Hatada S, Kondo S, Karube F, Kawaguchi Y (2007). Neocortical Inhibitory Terminals Innervate Dendritic Spines Targeted by Thalamocortical Afferents.. J Neurosci.

[85] Grafton ST, Hamilton AF (2007). Evidence for a distributed hierarchy of action representation in the brain.. Hum Mov Sci.

[86] Kakei S, Hoffman DS, Strick PL (2003). Sensorimotor transformations in cortical motor areas.. Neuroscience Res.

[87] Afraimovich V, Tristan I, Huerta R, Rabinovich MI (2008). Winnerless competition principle and prediction of the transient dynamics in a Lotka-Volterra model.. Chaos.

[88] Rabinovich M, Huerta R, Laurent G (2008). Neuroscience. Transient dynamics for neural processing.. Science.

[89] Kiebel SJ, Daunizeau J, Friston KJ (2009). Perception and hierarchical dynamics.. Front Neuroinform.

[90] Toussaint M, Gienger M, Goerick C (2007). Optimization of sequential attractor-based movement for compact behaviour generation..

[91] Müller JR, Philiastides MG, Newsome WT (2005). Microstimulation of the superior colliculus focuses attention without moving the eyes.. Proc Natl Acad Sci U S A.

[92] Wu W, Hatsopoulos NG (2007). Coordinate system representations of movement direction in the premotor cortex.. Experimental Brain Res.

[93] Takada M, Li K, Hattori T (1988). Dopaminergic nigrotectal projection in the rat.. Brain Res.

[94] Campbell KJ, Takada M (1989). Bilateral tectal projection of single nigrostriatal dopamine cells m the rat.. Neuroscience.

[95] Campbell KJ, Takada M, Hattori T (1991). Co-localization of tyrosine hydroxylase and glutamate decarboxylase in a subpopulation of single nigrotectal projection neurons.. Brain Res.

[96] Fuster JM (2001). The prefrontal cortex – an update: time is of the essence.. Neuron.

[97] Koechlin E, Ody C, Kouneiher F (2003). The architecture of cognitive control in the human prefrontal cortex.. Science.

[98] Kiebel SJ, Daunizeau J, Friston K (2008). A hierarchy of time-scales and the brain.. PLoS Comput Biol.

[99] Badre D (2008). Cognitive control, hierarchy, and the rostro-caudal 0rganization of the frontal lobes.. Trends Cogn Sci.

[100] Harrison LM, Bestmann S, Rosa MJ, Penny W, Green GGR (2011). Time scales of representation in the human brain: weighing past information to predict future events.. Front Hum Neurosci.

[101] Kojima S, Goldman-Rakic PS (1982). Delay-related activity of prefrontal neurons in rhesus monkeys performing delayed response.. Brain Res.

[102] Bick C, Rabinovich MI (2009). Dynamical origin of the effective storage capacity in the brain's working memory.. Phys Rev Lett.

[103] Lees AJ, Smith E (1983). Cognitive deficits in the early stages of Parkinson's disease.. Brain.

[104] Owen AM, James M, Leigh PN, Summers BA, Marsden CD (1992). Fronto-striatal cognitive deficits at different stages of Parkinson's disease.. Brain.

[105] Kwak Y, Muller MLTM, Bohnen NI, Dayalu P, Seidler RD (2010). Effect of Dopaminerigc Mediations on the Time Course of Explicit Motor Sequence Learning in Parkinson's Disease.. J Neurophys.

[106] Peterson DA, Elliott C, Song DD, Makeig S, Sejnowski TJ (2009). Probabilistic reversal learning is impaired in Parkinson's disease.. Neuroscience.

[107] Brown RG, Marsden CD (1988). Internal and external cues and the control of attention in Parkinson's disease.. Brain.

[108] Guitart-Masip M, Beierholm UR, Dolan R, Duzel E, Dayan P (2011). Vigor in the Face of Fluctuating Rates of Reward: An Experimental Examination.. J Cogn.

[109] Cools R, Barker RA, Sahakian BJ, Robbins TW (2003). L-Dopa medication remediates cognitive inflexibility, but increases impulsivity in patients with Parkinson's disease.. Neuropsychologia.

[110] Gotham AM, Brown RG, Marsden CD (1988). ‘Frontal’ cognitive function in patients with Parkinson's disease ‘on’ and ‘off’ levodopa.. Brain.

[111] Moustafa AA, Sherman SJ, Frank MJA (2008). dopaminergic basis for working memory, learning and attentional shifting in Parkinsonism.. Neuropsychologia.

[112] Frank MJ, Scheres A, Sherman SJ (2007). Understanding decision-making deficits in neurological conditions: insights from models of natural action selection.. Philos Trans R Soc Lond B Biol Sci.

[113] Doya K (2002). Metalearning and neuromodulation.. Neural Netw.

[114] Humphries MD, Wood R, Gurney K (2009). Dopamine-modulated dynamic cell assemblies generated by the GABAergic striatal microcircuit.. Neural Netw.

[115] Ahmed SHGM, Gutkin B (2009). Computational approaches to the neurobiology of drug addiction.. Pharmacopsychiatry.

[116] Hazy TE, Frank MJ, O'Reilly RC (2010). Neural mechanisms of acquired phasic dopamine responses in learning.. Neurosci Biobehav Rev.

[117] Moustafa AA, Gluck MA (2011). A neurocomputational model of dopamine and prefrontal-striatal interactions during multicue category learning by Parkinson patients.. J Cogn Neurosc.

[118] Daw ND, Gershman SJ, Seymour B, Dayan P, Dolan RJ (2011). Model-based influences on humans' choices and striatal prediction errors.. Neuron.

[119] Parush N, Tishby N, Bergman H (2011). Dopaminergic Balance between Reward Maximization and Policy Complexity.. Front Syst Neurosci.

[120] Mathys C, Daunizeau J, Friston KJ, Stephan KE (2011). A Bayesian foundation for individual learning under uncertainty.. Front Hum Neurosci.

[121] Potjans W, Morrison A, Diesmann M (2009). A spiking neural network model of an actor-critic learning agent.. Neural Comput.

[122] Deco G, Rolls ET, Romo R (2010). Synaptic dynamics and decision making.. Proc Natl Acad Sci U S A.

[123] Wanjerkhede SM, Bapi RS (2011). Role of CAMKII in reinforcement learning: a computational model of glutamate and dopamine signaling pathways.. Biol Cybern.

[124] Chevalier G, Deniau JM (1990). Disinhibition as a basic process in the expression of striatal functions.. Trends Neurosci.

[125] Ungerleider LG, Mishkin M, Ingle D, Goodale MA, Mansfield RJW (1982). Two cortical visual systems.. Analysis of Visual Behavior.

[126] Rosell A, Giménez-Amaya JM (1999). Anatomical re-evaluation of the corticostriatal projections to the caudate nucleus: a retrograde labeling study in the cat.. Neurosci Res.

[127] Gerfen CR, Wilson CJ, Swanson LW, Bjorklund A, Hökfelt T (1996). The basal ganglia. In: Handbook of Chemical Neuroanatomy.. Vol. 12: Integrated Systems of the CNS, Part III.

[128] Kravitz AV, Freeze BS, Parker PR, Kay K, Thwin MT (2010). Regulation of parkinsonian motor behaviours by optogenetic control of basal ganglia circuitry.. Nature.

[129] Crittenden JR, Graybiel AM (2011). Basal Ganglia disorders associated with imbalances in the striatal striosome and matrix compartments.. Front Neuroanat.

[130] Matsumoto M, Hikosaka O (2009). Two types of dopamine neuron convey positive and negative motivational signals.. Nature.

[131] Zweifel LS, Fadok JP, Argilli E, Garelick MG, Jones GL (2011). Activation of dopamine neurons is critical for aversive conditioning and prevention of generalised anxiety.. Nat Neurosci.

[132] Bromberg-Martin ES, Hikosaka O (2009). Midbrain Dopamine Neurons Signal Preference for Advance Information about Upcoming Rewards.. Neuron.

[133] Margolese HC, Chouinard G, Kolivakis TT, Beauclair L, Miller R (2005). Tardive dyskinesia in the era of typical and atypical antipsychotics. Part 1: pathophysiology and mechanisms of induction.. Can J Psychiatry.

[134] Bird E (1980). Chemical Pathology of Huntington's Disease.. Ann Rev Pharmacol Toxicol.

[135] Leckman JF, Bloch MH, Smith ME, Larabi D, Hampson M (2010). Neurobiological substrates of Tourette's disorder.. J Child Adolesc Psychopharmacol.

[136] Shannon KM (2005). Hemiballismus.. Curr Treat Options Neurol.

[137] Bezard E, Brotchie JM, Gross CE (2001). Pathophysiology of levodopa-induced dyskinesia: potential for new therapies.. Nat Rev Neurosci.

[138] Garrido MI, Kilner JM, Stephan KE, Friston KJ (2009). The mismatch negativity: a review of underlying mechanisms.. Clin Neurophysiol.

[139] Friston KJ, Kiebel SJ (2009). Predictive coding under the free-energy principle.. Phil Trans R Soc B.

[140] Friston K, Ao P (2012). Free-energy, value and attractors..

